# Radiomics for residual tumour detection and prognosis in newly diagnosed glioblastoma based on postoperative [^11^C] methionine PET and T1c-w MRI

**DOI:** 10.1038/s41598-024-55092-8

**Published:** 2024-02-25

**Authors:** Iram Shahzadi, Annekatrin Seidlitz, Bettina Beuthien-Baumann, Alex Zwanenburg, Ivan Platzek, Jörg Kotzerke, Michael Baumann, Mechthild Krause, Esther G. C. Troost, Steffen Löck

**Affiliations:** 1 OncoRay – National Center for Radiation Research in Oncology, Faculty of Medicine and University Hospital Carl Gustav Carus, Technische Universität Dresden, Helmholtz-Zentrum Dresden - Rossendorf, Dresden, Germany; 2 German Cancer Consortium (DKTK) Partner Site Dresden, Germany, and German Cancer Research Center (DKFZ), Heidelberg, Germany; 3 Division of Radiooncology/Radiobiology, German Cancer Research Center (DKFZ), Heidelberg, Germany; 4 National Center for Tumor Diseases (NCT), Partner Site Dresden, Germany: German Cancer Research Center (DKFZ), Heidelberg, Germany, Faculty of Medicine and University Hospital Carl Gustav Carus, Technische Universität Dresden, Dresden, Germany, and Helmholtz Association/Helmholtz-Zentrum Dresden - Rossendorf (HZDR), Dresden, Germany; 5 Department of Radiotherapy and Radiation Oncology, Faculty of Medicine and University Hospital Carl Gustav Carus, Technische Universität Dresden, Dresden, Germany; 6 Department of Nuclear Medicine, Faculty of Medicine and University Hospital Carl Gustav Carus, Technische Universität Dresden, Dresden, Germany; 7 Department of Radiology, German Cancer Research Center (DKFZ), Heidelberg, Germany; 8 Institute of Radiology, Faculty of Medicine and University Hospital Carl Gustav Carus, Technische Universität Dresden, Dresden, Germany; 9 Helmholtz-Zentrum Dresden-Rossendorf, Institute of Radiooncology, Dresden, Germany

**Keywords:** Machine learning, Prognostic markers, CNS cancer

## Abstract

Personalized treatment strategies based on non-invasive biomarkers have potential to improve patient management in patients with newly diagnosed glioblastoma (GBM). The residual tumour burden after surgery in GBM patients is a prognostic imaging biomarker. However, in clinical patient management, its assessment is a manual and time-consuming process that is at risk of inter-rater variability. Furthermore, the prediction of patient outcome prior to radiotherapy may identify patient subgroups that could benefit from escalated radiotherapy doses. Therefore, in this study, we investigate the capabilities of traditional radiomics and 3D convolutional neural networks for automatic detection of the residual tumour status and to prognosticate time-to-recurrence (TTR) and overall survival (OS) in GBM using postoperative [^11^C] methionine positron emission tomography (MET-PET) and gadolinium-enhanced T1-w magnetic resonance imaging (MRI). On the independent test data, the 3D-DenseNet model based on MET-PET achieved the best performance for residual tumour detection, while the logistic regression model with conventional radiomics features performed best for T1c-w MRI (AUC: MET-PET 0.95, T1c-w MRI 0.78). For the prognosis of TTR and OS, the 3D-DenseNet model based on MET-PET integrated with age and MGMT status achieved the best performance (Concordance-Index: TTR 0.68, OS 0.65). In conclusion, we showed that both deep-learning and conventional radiomics have potential value for supporting image-based assessment and prognosis in GBM. After prospective validation, these models may be considered for treatment personalization.

## Introduction

The standard of care in newly diagnosed glioblastoma multiforme (GBM) is maximal surgical resection, subsequent concurrent chemoradiation (RCT) with temozolomide followed by maintenance temozolomide for 6 months^1,2^. Despite the multimodal treatment, patients with GBM face an overall poor prognosis with a high recurrence rate and 5-year survival of less than 10%^3,4^. Gross total resection of GBM has been associated with improved local control and survival compared to subtotal or partial resection^5–7^. However, due to infiltrative growth patterns, total resection cannot always be achieved, and residual tumour cells persist after resection, leading to tumour recurrence and poor prognosis^8^.

In addition to the extent of resection, several factors that impact the survival in GBM have been identified including age, O6-methylguanine–DNA methyltransferase (MGMT) promoter methylation status, isocitrate dehydrogenase (IDH), and Karnofsky performance status (KPS) among others^9–11^. Many studies have performed gene-expression profiling to identify genes whose expression can predict patient survival in GBM^12–14^. However, none of these markers are currently used in clinical routine for personalized treatment approaches and reliable biomarkers are urgently needed^11^.

Post-surgical examination of GBM, including the assessment of the residual tumour status, is mainly based on gadolinium-diethylenetriaminepentaacetic acid (Gd-DTPA) enhanced T1c-w MRI. However, the reliability of T1c-w MRI alone in distinguishing tumour tissue from, inflammatory reparative changes after surgery is limited, and it suffers from high interindividual variability in target delineation for treatment planning^15,16^. Studies have shown that post-surgical amino acid positron-emission tomography (PET) such as L-[methyl-^11^C] methionine (MET) and O-(2-[18F]fluoroethyl)-L-tyrosine (FET) PET, respectively, has superior diagnostic value compared to T1c-w MRI as it can differentiate treatment-related changes from residual tumour progression with higher accuracy^17,18^. Concurrent PET/MRI offers great potential for the detection of residual tumour and guided therapy intensification for treatment personalization^18,19^.

In recent years, conventional radiomics and deep learning (DL)-based radiomics have been widely used as non-invasive methods for computer assisted diagnosis and prognosis in various cancer entities^20–23^. Conventional radiomics extracts and analyses handcrafted features from medical imaging data, while (DL)-based radiomics uses deep neural networks such as convolutional neural networks (CNNs) to perform the same task. Currently, only few studies have evaluated automatic detection of residual tumours. Zeng et al.^24^ and Miere et al.^25^ have reported an auto-segmentation method for the GBM residual tumour volume on T1c-w MRI. For prognostic modelling in GBM, various studies have evaluated conventional radiomics using multiparametric MRI (mpMRI) to evaluate overall survival (OS) and progression free survival (PFS)^26–29^. Integrating pre-treatment MRI radiomics features with clinical and molecular features was shown to further improve the prognostic performance^30^. Recently, a study by Garcia‑Ruiz et al.^31^ showed the high correlation of radiomics features extracted from the enhancing residual tumour region on early post-surgical MRI with OS (AUC 0.71). Only a limited number of studies have conducted radiomics analyses to assess the prognostic significance of FET-PET imaging in GBM^32,33^. However, these investigations were specifically based on post-RCT PET images.

To the best of our knowledge, comparative analysis of conventional feature-based and DL-based radiomics has not yet been performed for the detection of residual tumours and to evaluate the prognostic role of pre-RCT MET-PET/MRI in patient with GBM. The recently published dataset of the prospective PETra trial^18^ is well suited for that task since it contains both imaging modalities. Therefore, in this study we develop and independently validate conventional and DL-based radiomics models to identify the residual tumour status and prognosticate TTR and OS in newly diagnosed GBM patients using MET-PET and T1c-w MRI.

## Materials and methods

### Patient data

Imaging and clinical data of 132 adult patients with GBM was used, originating from the PETra trial, which is a prospective one-arm, single-centre, nonrandomized biomarker study as previously described (85 patients; clinicaltrials.gov; NCT01873469)^18^, and from an additional retrospective validation cohort (47 patients). All patients were newly diagnosed with histologically confirmed GBM and were treated at the University Hospital and Faculty of Medicine Carl Gustav Carus, Dresden, Germany. All 85 patients from the PETra trial (ethics id. EK-41022013) were allocated to the training data, and the 47 consecutive patients from validation trial (ethics id. EK-390072021) were allocated to the independent test data. All patients gave written informed consent. The study was approved by the ethics committee of TU Dresden (EK-41022013, EK-390072021) and was conducted in accordance with the relevant guidelines and regulations, i.e. the Declaration of Helsinki, version 2013. Patients underwent standard RCT with temozolomide and radiotherapy dose of 60 Gy in 2 Gy-fractions, starting within 7 weeks after surgery. The inclusion criteria for this retrospective radiomics analysis were: T1c-w MRI acquired contemporaneously with MET-PET before RCT with sufficient imaging quality, i.e. absence of strong artifacts, and availability of the considered endpoints.

### Image acquisition, endpoints, and contouring

The PET/MRI investigations were carried out on a 3 Tesla Ingenuity TF PET/MRI scanner (Philips Healthcare, Best, The Netherlands). For post-surgical MRI, the T1c-w sequence was utilized for this study. Image acquisition details for MET-PET and T1c-w MRI of training and test data are summarized in Supplementary Table S1 and in^18^. The considered endpoints were the residual tumour status individually assessed on MET-PET and T1c-w MRI, and time-to-recurrence (TTR), and overall survival (OS) using both imaging modalities.

To determine the presence of residual tumour after surgery, qualitative evaluation of MET-PET and T1c-w MRI data was performed individually to establish binary ground truth labels. A nuclear medicine expert (B.B.) evaluated the reconstructed MET-PET images acquired 20–40 min after tracer injection using the ROVER software package (ABX). Residual tumour status on MET-PET was labelled as positive (1) if there were focal uptake areas representing the presence of true residual tumours without physiologically enhanced uptake or enhancement in postsurgical alteration. The residual tumour status for T1c-w MRI was evaluated by a radiation oncologist (A.S.) using early postsurgical T1c-w MRI in combination with operative reports and the second baseline T1c-w MRI, acquired contemporaneously with MET-PET (used in this analysis; acquisition median 23 days after surgery). If the second T1c-w MRI showed no residual tumour, the residual T1c-w MRI status was set as negative (0). In case of distinct progression between the two T1c-w MRI scans, the status was changed to positive (1). Difficult cases with small residual tumours or laminar enhancement, hampering the clear distinction from residual blood in the cavity, were independently reviewed by an experienced radiologist (I.P.). A more detailed description of the qualitative analysis performed by human raters to evaluate residual tumour status is given in^18^.

The survival endpoints TTR and OS were calculated from the first day of RCT to the day of the event (local recurrence for TTR and death for OS) or censoring. For the patients with the observed event, the event time was accompanied by an event indicator variable of 1, whereas for patients without an event, the last follow-up time was used together with an event indicator variable of 0.

PET/MRI images were co-registered to the treatment planning computed tomography (CT) using the treatment planning system RayStation 8B SP2 (RaySearch Laboratories, Stockholm, Sweden) and the clinical target volume (CTV) was transferred to MET-PET and T1c-w MRI.

### Study design

**Figure 1:**
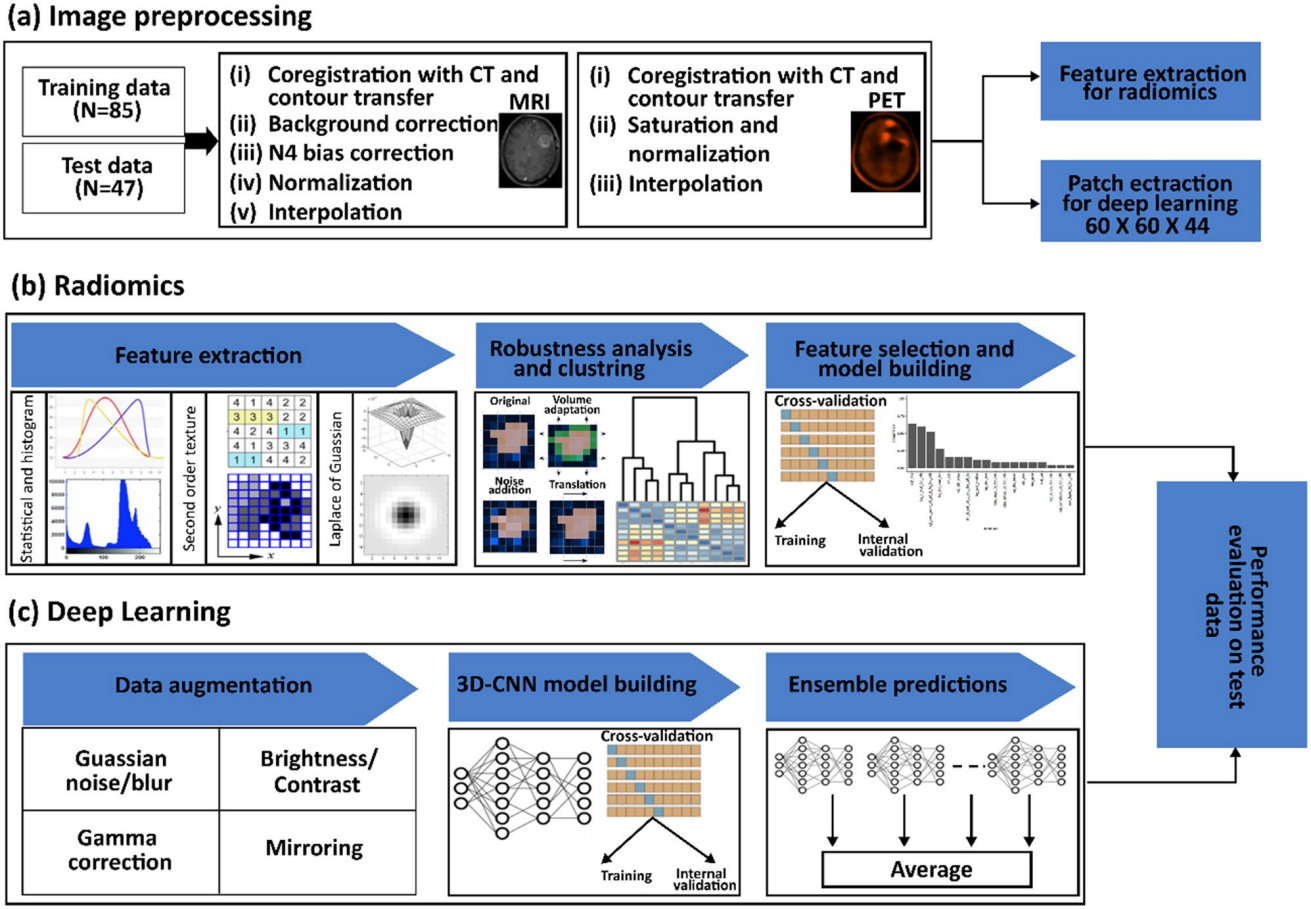
Study design. (**a**) Image preprocessing. (**b**) Radiomics features were extracted from each imaging modality, analysed for robustness, and clustered. Radiomics signatures for MET-PET and T1c-w MRI were developed in a cross-validation approach and applied to the test data. (**c**) 3D-CNN models were trained in a cross-validation approach. Subsequently, the performance of ensemble predictions was evaluated on the test data.

We developed and independently validated conventional radiomics and DL-based signatures for predicting the residual tumour status and for the prognosis of TTR and OS in patients with GBM based on MET-PET and T1c-w MRI data acquired before RCT. Figure 1 summarizes the design of this study.

For the conventional radiomics analysis, we utilized the CTV to separately compute imaging features in MET-PET and T1c-w MR imaging. These features included first-order features, second-order texture features, and Laplacian of Gaussian (LoG) transformed intensity features. The features were filtered for stability under small image perturbations and clustered. Separate radiomics models were developed using the training data (N = 85) for each imaging modality and independently validated on the test data (N = 47). In our DL-based radiomics analysis, three different 3D-CNN architectures were used, i.e. 3D-VGGNet, 3D-Resnet, and 3D-DenseNet. 3D-CNN models were trained from scratch on image patches extracted around the CTV centre of mass individually for each imaging modality. Training was performed using two approaches, without data augmentation and with data augmentation. We then applied the developed models to independent test data and compared their performance. The final predictions from both approaches for the prognosis of TTR and OS were integrated with important clinical/molecular features in multivariable Cox regression, which was then validated on the test dataset. We describe image processing and modelling in more detail in the following paragraphs.

### Image pre-processing, and feature extraction

The pre-processing steps used for conventional radiomics and DL-modelling are depicted in Fig. 1a. For both analyses, T1c-w MR imaging was subjected to bias correction using the N4ITK algorithm^34^ and soft tissue masking via the Canny Edge detection algorithm^35^. After bias correction, z-score normalization was applied to the intensity values of T1c-w MRI within the soft tissue mask. PET imaging was converted to (body-weight) Standardized Uptake Values (SUV), and SUV values outside the [0,10] range were truncated to remove potential outliers. Finally, the entire volume was normalized to the [0,1] range to standardize the data.

Further pre-processing was specific to conventional radiomics or DL-based radiomics analysis. For the DL-based radiomics analysis, we aligned the orientation of MET-PET and T1c-w MR images and resampled these to isotropic 2.0 × 2.0 × 2.0 mm^3^ voxels using trilinear interpolation. A single image volume of size 60 × 60 × 44, centred around the CTV centre of mass was extracted in the axial plane for both imaging modalities.

For the conventional radiomics analysis, further image pre-processing followed by feature extraction was carried out using the MIRP Python toolkit (version 1.1.3)^36^. MET-PET and T1c-w MR image voxels were resampled to 2.0 × 2.0 × 2.0 mm^3^ and 1.0 × 1.0 × 1.0 mm^3^, respectively, using trilinear interpolation. LoG filters with kernel widths σ = 2 mm for MET-PET and σ = 1 mm for T1c-w MRI were applied to the base images. The choice of kernel width was based on the original slice thickness of each imaging modality. A total of 270 and 152 intensity-based and texture-based features were extracted from the 3D CTV on the baseline MET-PET and T1c-w MRI, respectively. In addition, 57 first-order intensity-based features were extracted from the CTV on the LoG transformed images for both imaging modalities. This resulted in a total of 327 and 209 features extracted from MET-PET and T1cw-MRI, respectively. Further details on feature classes are summarized in Supplementary Table S2. Image pre-processing and feature extraction in MIRP were implemented according to the recommendations of the Image Biomarker Standardization Initiative (IBSI)^37^. The definitions used to calculate the features can be found in the IBSI reference manual. Image processing parameters are summarized in Supplementary Table S3.

To ensure reproducible results in radiomics analysis, the imaging features should remain stable under minor perturbations, such as slight variations in acquisition parameters or positioning uncertainties^36^. To assess feature robustness, we performed the following image augmentations on the training data: adding Gaussian noise (mean 0, standard deviation equal to that in the image), random volume changes of the CTV (0%, − 15%, 15%), and translations (0.0, 0.25, and 0.75 mm) in all three spatial dimensions. All combinations of these perturbations were considered, leading to 81 perturbed images for each original dataset. The intra-class correlation coefficient (ICC) was calculated with a 95% confidence interval, quantifying the similarity of feature values under different perturbations for every feature. Features with the lower boundary of the 95% confidence interval of the ICC below 0.8 were removed^36^.

Feature redundancy was lessened through clustering of highly similar features. The Spearman correlation coefficient ($\rho$) was used as a similarity metric with average linkage as a criterion for merging two clusters; $\rho \ge$ 0.8 was defined for placing features into the same cluster. The feature with the highest mutual information with the endpoint was selected as the representative for each cluster. The clustering process was done separately for MET-PET and T1c-w MRI-based feature sets.

### Conventional radiomics modelling

Figure 1b illustrates the workflow for conventional radiomics analysis. We implemented a workflow containing four major processing steps to derive radiomics signatures from the pre-processed feature sets: (i) feature pre-processing, (ii) feature selection, (iii) model building with internal validation, and (iv) testing. This workflow was implemented using the open-source end-to-end statistical learning software package familiar (1.0.0)^38^ in R (version 4.0.3).

Steps (i)–(iii) were first performed using 5 repetitions of fivefold stratified cross-validation (CV) nested in the training dataset to identify an optimal signature, i.e. the steps were repeatedly performed on the internal training part and validated on the internal validation part of the CV folds. After identifying the final signature, a final model was developed on the entire training data and validated on the test dataset. The following procedure was performed for each of the 25 CV runs:

- Features were transformed using the Yeo-Johnson transformation to align their distribution to a normal distribution. Afterwards, features were z-transformed to mean zero and standard deviation one. Both transformations were performed on the internal training part and applied unchanged to the features of the internal validation part.
- Four supervised feature-selection algorithms were considered: minimal redundancy maximum relevance (MRMR)^39^, mutual information maximization (MIM)^40^, elastic-net (EN)^41^, and univariate regression (UR)^42^. To avoid potential overfitting, only the five most relevant features were selected in each CV fold.
- The selected features were used by three different classifiers: logistic regression (GLM_logistic), random forest (RF), and Xgboost linear model (XGB_lm) for the detection of residual tumour status and Cox regression (Cox), random survival forest (RSF), and XGB_lm for prognosis of TTR and OS. Model hyperparameters were tuned automatically using a variant of the sequential model-based optimisation (SMBO)^43^ algorithm based on a bootstrap sampling of the training data. Each classifier was built on the internal training part, which was validated on the internal validation part.

After cross-validation, features were ranked according to their occurrence across the 25 CV folds for each of the feature-selection methods. The top 5 most commonly occurring features that appeared in at least 75% (i.e. 3 out of 4) of feature-selection methods were selected. If a subset of these features showed a Spearman correlation ρ > 0.5 with each other on the entire training data, the most relevant feature was considered, i.e. the one showing higher association with endpoint on the training data. A detailed example of the feature-selection scheme for the detection of residual tumour status and prognosis of TTR using MET-PET imaging is presented in Supplementary Section 1: Tables S4, S5 and Fig. S1a,b, respectively. The resulting radiomics signature was then used to build a classification or survival model using the entire training dataset.

### Deep-learning-based radiomics

Three different 3D-CNN models, i.e. 3D-VGGNet, 3D-ResNet, and 3D-DenseNet, were trained from scratch in our DL-based radiomics analysis. Model architectures were adapted to get the best performance on the internal validation data.

The 3D-VGGNet model consist of 3 convolution blocks with 2 convolution layers in first two blocks and 3 convolution layers in third block (filter size = 3 × 3 × 3, activation = ReLU) followed by max-pooling (pool-size = 2 × 2 × 2) and dropout layer (rate = 0.4). The first block comprised 64 filters. The number of filters was doubled in each subsequent block. A batch normalization and flattening operation followed the last convolutional block. The 3D-ResNet network was based on a vanilla ResNet18 implementation for 3D image data adapted from^44^. The first convolutional layer was modified to use a filter size of 3 × 3 × 3, a stride of 2 and global average pooling after the last residual block followed by the flattening layer. The 3D-DenseNet121 network was adapted from^45^. Instead of using 4 dense blocks as in the original DenseNet implementation^46^, only 3 dense blocks (6, 12, 24 layers per block) were used. Like the 3D-ResNet18 adaptation we used a filter size of 3 × 3 × 3 with a stride of 2 in the first convolution layer and global average pooling after the last residual block followed by a flattening layer.

To further enhance the performance of the architectures mentioned above, they were modified by adding a group of four fully connected (FC) layers with 512, 512, 256, and 128 neurons at the end of the network. To prevent overfitting, a dropout rate of 0.4 was applied between these FC layers. The model's output was determined by a single dense neuron with tanh activation. Overview of the 3D-CNN architectures utilized in this study is presented in Supplementary Figs. S2 and S3.

For model training, we employed an Adam optimizer and a batch size of 16. The training process employed a maximum of 300 epochs with early stopping (patience = 100) and an adaptive learning rate that utilized exponential decay (initial learning rate = 1.10^–4^, decay steps = 1000, decay rate = 0.96) through Keras callbacks. To optimize model losses for detecting residual tumour status, binary cross-entropy (BCE) loss was used, a commonly used loss function for binary classification problems^47^. Model losses for the TTR and OS endpoints were optimized using a survival-specific loss function, i.e. Cox proportional hazard model (CPHM) similar to previous work^23,48,49^. The CPHM minimizes the negative of the Cox partial log-likelihood function to estimate log-hazard values for each batch of imaging data, which were then transformed by tanh activation to restricted hazard output within the range of (exp(− 1), exp(1)).

For the analysis of each endpoint, network training was performed within 5 repetitions of fivefold cross-validation (CV), stratified by the event status, on the training dataset. For each of the CV folds, training volumes were augmented by changing contrast, brightness, Gamma correction, Gaussian noise, and Gaussian blur using the open-source batchgenerators python package for data augmentation^50^. Details regarding data augmentation parameters are provided in Supplementary Section 2 and Supplementary Table S6. Model training was performed on the training split of the CV folds and model losses were evaluated at the end of each epoch on the internal validation fold. Since each of the 25 CV runs resulted in a trained model, an ensemble prediction was created by averaging outputs for each patient. Training ensemble prediction was obtained by averaging the predicted output for each patient across the 20 models for which that patient was part of the training fold. Similarly, internal validation ensemble prediction was computed by averaging the predicted output using the remaining five models for which the patient was assigned to the internal test fold. All trained 25 models were then applied to independent test data and a patient’s ensemble prediction was computed by averaging over all 25 model predictions. To assess the benefit of data augmentation on model generalization to unseen data, the above pipeline was also implemented without augmenting the training data.

### Combination with clinical data

Finally, in order to create joint clinical and imaging signatures for TTR and OS prognosis, clinical/molecular features that were significantly associated to TTR and OS in univariable Cox regression were first used to create a stand-alone clinical signature using multivariable Cox regression. Then, we integrated these clinical features with the selected radiomic signature and with the 3D-CNN ensemble prediction. In order to avoid correlation between image-based and clinical features, we calculated the Spearman correlation between them and retained only those features with a correlation of < 0.5. Finally, a multivariable Cox model was fitted on the training data, and then applied to the test data.

### Statistical analysis

The following baseline clinical parameters were available: gender, age, ECOG status, MGMT promoter methylation status, IDH mutation status, and resection type. Categorical clinical features were compared between training and test data by the Chi-squared (χ^2^) test whereas continuous features were compared using the Mann–Whitney-*U* test. Available clinical features were associated with TTR and OS by univariable Cox regression.

Associations between the final model predictions and the endpoints were evaluated by the AUC for the detection of residual tumour status and by the C-index for the prognosis of TTR and OS. The estimated value and the 95% confidence interval of these metrics were computed. For creating a confusion matrix based on the final radiomics and DL prediction for residual tumour status classification, an optimal cutoff was selected on the training data using the Youden index and transferred to the internal validation and independent test data. For association with TTR and OS, patients were stratified into low and high-risk groups using an optimal cutoff on the training data that was based on maximally selected rank statistics^51^. The cutoff was transferred to the internal validation and independent test data and TTR and OS of stratified groups were assessed with Kaplan Meier curves compared with the log-rank test.

Model calibration was assessed via the Hosmer–Lemeshow goodness of fit test (HL test)^52^ for the prediction of residual tumour status and the Greenwood Nam d’Agostino test (GND test)^53^ for TTR and OS respectively, and by creating calibration plots. Correlations between features were assessed by the Spearman correlation coefficient ($\rho$). All tests were two-sided with a significance level of 0.05. The importance of individual features in the final signature was assessed through univariate fitting of a logistic regression model (residual tumour status) or Cox regression (TTR and OS) and computing Wald-test *p*-values.

Conventional radiomics analysis was performed in R version 4.0.3, while DL-based radiomics analysis was performed in Python 3.7.0 and Keras (v2.3.1) with TensorFlow (v2.1.0) on NVIDIA GeForce RTX 2080 Max-Q. Our code is publicly available from https://github.com/oncoray/cnn-petra.

## Results

Patient characteristics of the training and test data are summarized and compared in Table 1. A significant difference between the two cohorts was observed for MGMT status and age. Patients in the training dataset had a lower percentage of methylated MGMT status (*p*-value 0.019), and a slightly lower median age (*p*-value 0.049) compared to the test data. In univariate Cox analysis, a significant association to TTR and OS was observed for MGMT status (OS: *p* < 0.001), age (TTR: *p* 0.034, OS: *p* 0.001) and IDH status (TTR: *p* 0.018) on the training data, as shown in Supplementary Table S7. Due to the small number of IDH mutated cases (N = 6), however, IDH status was not considered for the clinical signature.

**Table 1:** Patient, tumour, and treatment characteristics for the training and test data.

Variable		Training (85)		Test (47)		*p*-value
		Median	Range	Median	Range	
Age	Years	58	23–82	61	24–77	0.049
TTR	Months	7.43	0–73.0	9.76	1.15.58.0	0.60
OS	Months	16.6	1.54–73.0	13.9	1.94–58.0	0.10
		**Number**	**%**	**Number**	**%**	
Gender	Male/female	51/34	60.0/40.0	31/16	66.0/34.0	0.63
ECOG	0/1/2/unknown	45/35/5/0	52.9/41.2/5.9/0	21/19/3/4	44.7/40.4/6.4/8.5	0.054
MGMT	Wildtype/methylated/unknown	56/29/0	65.9/34.1/0	20/26/1	42.6/55.3/2.1	0.019
Resection	GTR/STR/BIO	49/29/7	57.6/34.1/8.2	26/21/0	55.3/44.7/0.0	0.09
IDH	Wildtype/mutated/unknown	75/6/4	88.2/7.1/4.7	44/2/1	93.6/4.3/2.1	0.60
PET status	0/1 (negative, positive)	28/57	32.9/67.1	17/30	36.2/63.8	0.85
MRI status	0/1 (negative, positive)	49/36	57.6/42.4	23/24	48.9/51.1	0.44
TTR status	0/1 (censored, event)	11/74	12.9/87.1	12/35	25.5/74.5	0.11
OS status	0/1 (censored, event)	13/72	15.3/84.7	17/30	36.2/63.8	0.011

BIO, biopsy; ECOG, Eastern Co-operative Oncology Group; GTR, gross total resection; IDH, isocitrate dehydrogenase; MGMT, O6-methylguanine DNA methyltransferase; MRI, magnetic resonance imaging; OS, overall survival; PET, positron emission tomography; STR, subtotal resection; TTR, Time-to-recurrence. Age was compared using Mann–Whitney-U test, TTR and OS were compared using log-rank test and Categorical variables were compared using χ2 test between training and test data.

Table 2 (top rows) presents the results for the classification of residual tumour status in MET-PET and T1c-w MRI using conventional radiomics, including model names and finally selected features. In internal CV, overall higher performance for the prediction of residual tumour status in MET-PET was observed for all considered machine learning models (AUC 0.93) compared to T1c-w MRI (AUC 0.66–0.68). Similarly, on the test data, a higher performance was observed for detection of residual tumour status in MET-PET (AUC 0.90–0.91) compared to T1c-w MRI (AUC 0.73–0.78). Overall, logistic regression model showed best performance compared to other machine learning methods for residual tumour detection in both imaging modalities. Corresponding confusion matrices for the logistic regression model are shown in Supplementary Fig. S4a with a sensitivity of 0.73 and 0.54 and a specificity of 0.88 and 0.87 on the test data for residual tumour status in MET-PET (threshold 0.77) and T1c-w MRI (threshold 0.38), respectively. The selected MET-PET feature was log_ih_kurt_fbn_n16 (IBSI: C317). It represents the kurtosis of the discretized histogram (16 bins) on the LoG transformed images. High values indicate the presence of high intensities within the CTV with pronounced peaks of MET uptake, which was related to a positive residual tumour status in comparison to the PET-negative group with relatively low values of this feature. The feature showed a significant contribution both in training and test (*p* < 0.01), box plots are presented in Supplementary Fig. S5. The definition of the selected features is given in Supplementary Table S8 and the logistic regression models for the best performing signatures are presented in Supplementary Table S9.

**Table 2:** Area under the curve (AUC) values for cross-validation (CV) and independent test data for residual tumour detection based on MET-PET and T1c-w MRI using conventional radiomics (top six rows) and deep learning (DL) radiomics (bottom six rows).

Modality	Model	CV train AUC	CV valid AUC	Features	Final training AUC	Final test AUC
MET-PET	**GLM logistic**	0.95 (0.88–0.99)	0.93 (0.60–1.00)	log_ih_kurt_fbn_n16	0.92 (0.86–0.97)	**0.91 (0.81–0.98)**
	RF	0.97 (0.90–1.00)	0.93 (0.57–1.00)	log_ih_kurt_fbn_n16	0.93 (0.87–0.97)	0.90 (0.80–0.97)
	XGB_lm	0.94 (0.87–0.99)	0.93 (0.58–1.00)	log_ih_kurt_fbn_n16	0.92 (0.86–0.97)	0.91 (0.81–0.98)
T1c-w MRI	**GLM logistic**	0.78 (0.62–0.90)	0.66 (0.22–0.97)	dzm_ldhge_3d_fbn_n32, ih_rmad_fbn_n32	0.76 (0.65–0.87)	**0.78 (0.64–0.89)**
	RF	0.87 (0.62–0.89)	0.68 (0.22–0.99)	dzm_ldhge_3d_fbn_n32, ih_rmad_fbn_n32	0.86 (0.78–0.94)	0.73 (0.58–0.87)
	XGB_lm	0.76 (0.74–0.98)	0.66 (0.24–0.99)	dzm_ldhge_3d_fbn_n32, ih_rmad_fbn_n32	0.77 (0.63–0.87)	0.78 (0.64–0.90)
MET-PET	**DenseNet**	1.00 (0.99–1.00)	0.96 (0.93–0.99)	–	–	**0.95 (0.89–1.00)**
	ResNet	1.00 (1.00–1.00)	0.92 (0.87–0.98)	–	–	0.81 (0.70–0.94)
	VGGNet	1.00 (1.00–1.00)	0.95 (0.90–1.00)	–	–	0.93 (0.86–1.00)
T1c-w MRI	DenseNet	1.00 (0.99–1.00)	0.77 (0.68–0.87)	–	–	0.63 (0.47–0.80)
	ResNet	1.00 (1.00–1.00)	0.73 (0.63–0.84)	–	–	0.61 (0.44–0.78)
	**VGGNet**	0.99 (0.98–1.00)	0.71 (0.59–0.82)	–	–	**0.71 (0.55–0.86)**

Values in parenthesis represent the 95% confidence interval. Best test performance is marked in bold.

**Figure 2:**
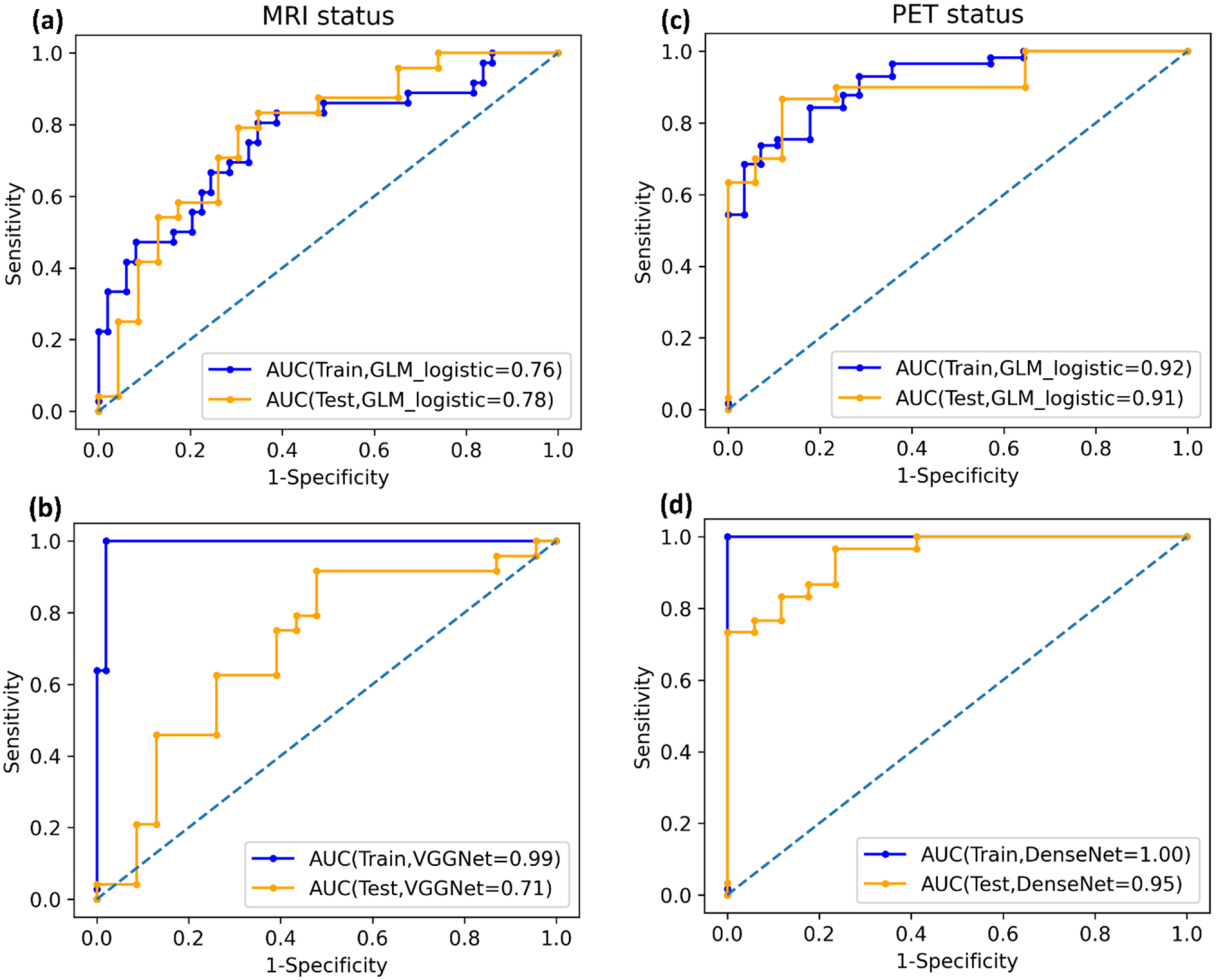
Receiver operating characteristics (ROC) curves of the best performing conventional radiomics and deep-learning-based model for classification of residual tumour status on (**a**, **b**) T1c-w MRI and (**c**, **d**) MET-PET in training and test data.

The same analysis was then repeated using 3D-CNNs. In general, 3D-CNNs trained with data augmentation showed higher performance in internal CV folds compared to 3D-CNN models trained without data augmentation for residual tumour detection as well as for the prognosis of TTR and OS (Supplementary Tables S10 and S11). Therefore, only models created with data augmentation were evaluated on the test data. Table 2 (bottom rows) presents the results of DL-based radiomics for predicting the residual status, including model names. In internal CV, DenseNet showed the highest AUC for both imaging modalities. As for conventional radiomics, detection of residual tumour status in MET-PET (AUC 0.92–0.96) was more accurate than in T1c-w MRI (AUC 0.71–0.77). On the test data, the highest performance was also achieved by DenseNet on MET-PET (AUC 0.95), while VGGNet showed a better performance for T1c-w MRI (AUC 0.71). Confusion matrices for best performing models are presented in Supplementary Fig. S4b showing a sensitivity of 0.97 and 0.38 and a specificity of 0.71 and 0.87 for MET-PET (threshold 0.56) and T1c-w MRI (threshold 0.40) based classification, respectively. Figure 2 shows the receiver operating characteristic (ROC) curves of the described models from conventional radiomics and DL. The corresponding calibration plots are shown in Supplementary Fig. S6.

Table 3 presents the results for the prognosis of TTR and OS using conventional radiomics. In internal CV, acceptable performance was only observed for TTR prediction based on MET-PET (C-index 0.58–0.61). This translated to the test cohort, where the signatures developed on MET-PET showed a better performance (C-index 0.58–0.59) than the signatures developed on T1c-w MRI (C-index 0.53–0.54). Furthermore, prediction of OS on the test cohort yielded acceptable results with T1c-w MRI (C-index 0.62–0.63). None of the models achieved significant stratification of patients in low and high-risk groups of OS on the test data (*p*-value > 0.05).

**Table 3:** Concordance index (C-index) for the endpoint time to recurrence (TTR) and overall survival (OS) based on MET-PET imaging and T1c-w MRI data using conventional radiomics.

Endpoint	Modality	Model	CV train C-index	CV valid C-index	Features	Final training C-index	Final test C-index	*p*-value test
TTR	MET-PET	Cox	0.66	0.60	log_stat_min	0.64 (0.57–0.71)	0.59 (0.48–0.70)	0.25
		RSF	0.64	0.58		0.64 (0.57–0.51)	0.58 (0.47–0.69)	0.23
		XGB_lm	0.66	0.61		0.64 (0.56–0.71)	0.59 (0.48–0.70)	0.25
	T1c-w MRI	Cox	0.62	0.51	ivh_diff_i25_i75, dzm_zd_var_3d_fbn_n32, loc_peak_glob	0.60 (0.52–0.67)	0.54 (0.42–0.64)	0.58
		RSF	0.64	0.53		0.64 (0.58–0.72)	0.53 (0.42–0.65)	0.89
		XGB_lm	0.62	0.51		0.59 (0.52–0.66)	0.54 (0.44–0.65)	0.23
	Clinical	Cox	–	–	Age, MGMT	**0.72 (0.65–0.79)**	**0.59 (0.49–0.71)**	**0.004**
	Clinical + MET-PET	Cox	–	–	Age, MGMT, log_stat_min	**0.74 (0.68–0.79)**	**0.66 (0.56–0.76)**	** < 0.001**
	Clinical + T1c-w MRI	Cox	–	–	Age, MGMT, ivh_diff_i25_i75, dzm_zd_var_3d_fbn_n32, loc_peak_glob	**0.74 (0.67–0.79)**	**0.62 (0.51–0.73)**	**0.008**
OS	MET-PET	Cox	0.63	0.52	stat_max	0.60 (0.53–0.68)	0.60 (0.46–0.74)	0.84
		RSF	0.65	0.51		0.60 (0.52–0.68)	0.60 (0.48–0.70)	0.85
		XGB_lm	0.63	0.54		0.60 (0.53–0.68)	0.60 (0.47–0.73)	0.84
	T1c-w MRI	Cox	0.62	0.49	ivh_diff_i25_i75, dzm_zd_var_3d_fbn_n32	0.60 (0.53–0.67)	0.63 (0.49–0.73)	0.86
		RSF	0.65	0.51		0.61 (0.53–0.70)	0.62 (0.48–0.74)	0.63
		XGB_lm	0.62	0.49		0.59 (0.52–0.66)	0.62 (0.50–0.73)	0.3
	Clinical	Cox	–	–	Age, MGMT	0.74 (0.69–0.81)	0.55 (0.45–0.66)	0.32
	Clinical + MET-PET	Cox	–	–	Age, MGMT, stat_max	0.75 (0.60–0.80)	0.59 (0.48–0.69)	0.21
	Clinical + T1c-w MRI	Cox	–	–	Age, MGMT, ivh_diff_i25_i75, dzm_zd_var_3d_fbn_n32	0.76 (0.70–0.81)	0.57 (0.46–0.67)	0.25

Values in parenthesis represent the 95% confidence interval. Best performance is marked in bold.

**Figure 3:**
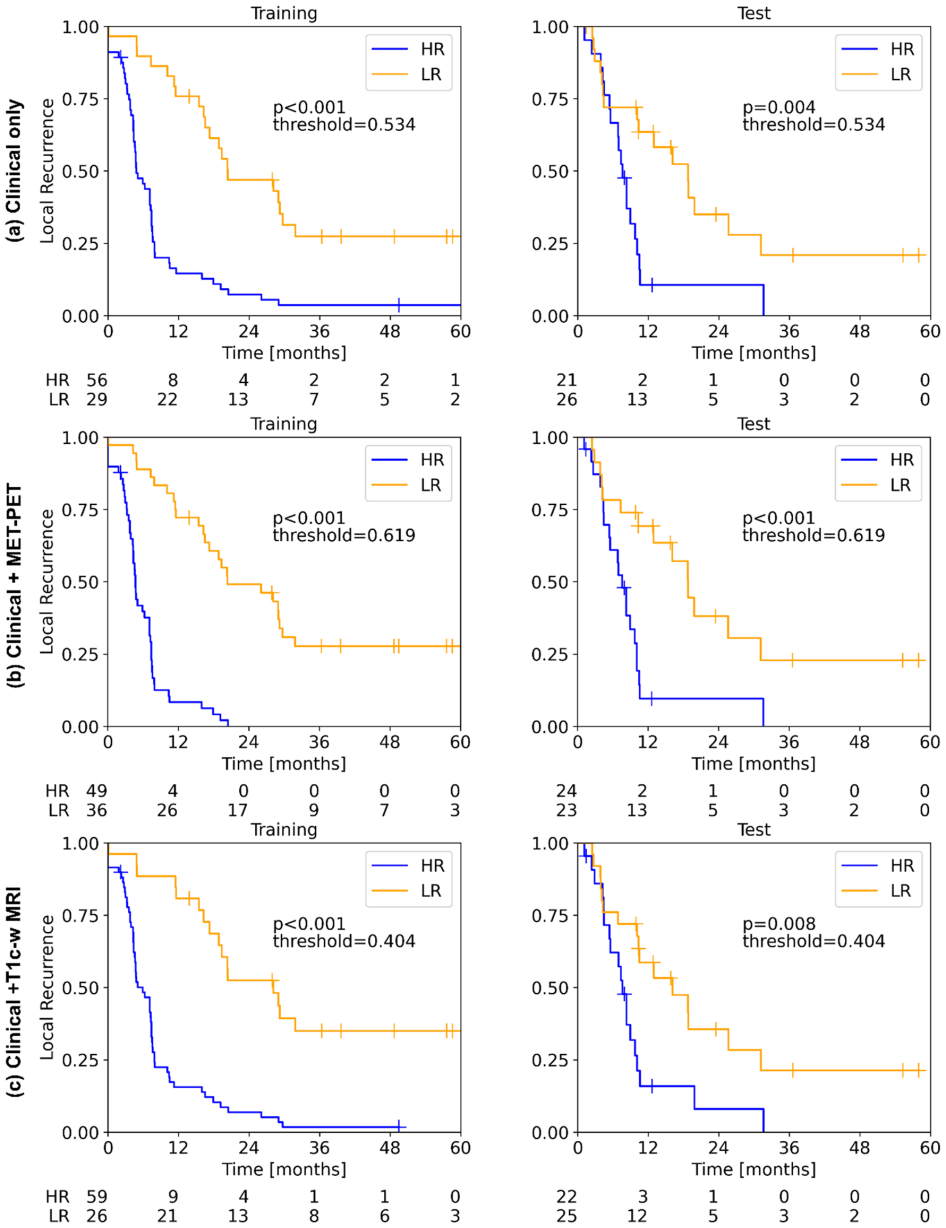
Kaplan–Meier plots for the prognosis of TTR on the training and test cohort using the Cox regression model based on (**a**) the clinical signature, (**b**) the clinical + MET-PET signature, and (**c**) the clinical + T1c-w MRI signature. Imaging-based signatures were developed using conventional radiomics. All models resulted in significant patient stratification into low and high-risk groups (*p* < 0.01) on the test set.

The Cox regression model containing clinical features (age and MGMT status) showed a decent performance for prognosis of TTR on the test data, with significant risk group stratification (C-index 0.59, *p* 0.004), while the performance for prognosis of OS was relatively low (C-index 0.55, *p* 0.32). Combining this clinical signature with imaging signatures showed improved prognostic performance with significant stratification of the patients into low and high-risk groups for TTR (clinical + MET-PET: C-index 0.66, *p* < 0.001; Clinical + T1cw-MRI: C-index 0.62, *p* 0.008), while for the prognosis of OS, the performance still remained low. Figure 3 shows the Kaplan–Meier curves for the clinical model (a) the clinical + MET-PET model (b), and the clinical + T1cw-MRI model (c) for prognosis of TTR. The corresponding calibration plots are shown in Supplementary Fig. S7a–c. Corresponding model and transformation parameters for the best performing signatures developed are presented in Supplementary Table S12.

The selected MET-PET features for the prognosis of TTR and OS were log_stat_min and stat_max (IBSI:1GSF), respectively. Both features are intensity-based statistical features that describe how intensities (or SUV values in case of MET-PET imaging) within the ROI are distributed. The highest SUV present within the CTV on baseline MET-PET is captured by the stat_max feature, which is closely related to the minimum SUV on LoG transformed MET-PET images. High values of stat_max and consequently low values of log_stat_min indicate MET uptake in the residual tumour. Image-based interpretation of these features is presented in Supplementary Fig. S8. Patients in the low-risk group of TTR showed relatively high values of log_stat_min, i.e. no high SUV present (a), while patients in the high risk group had lower log_stat_min, which translates to the existence of bright voxels or alternatively high values of stat_max in the CTV (b).

**Figure 4:**
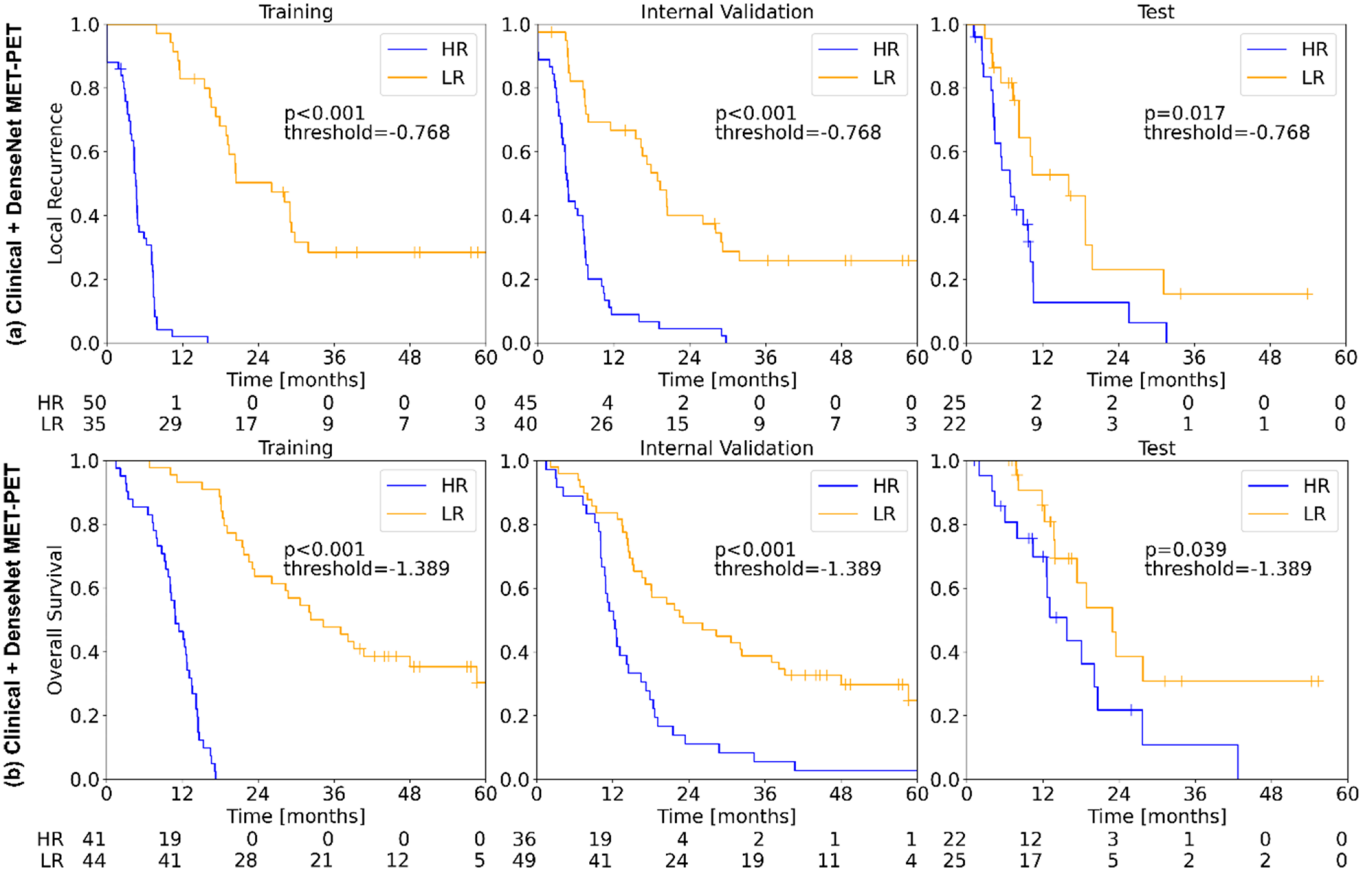
Kaplan–Meier estimates for risk-group stratification for (**a**) time to recurrence (TTR) and, (**b**) overall survival (OS) in training, internal validation and independent test data based on the respective joint clinical + ensemble predictions (3D-DenseNet model) on MET-PET data.

Table 4 presents the results for the prognosis of TTR and OS using 3D-CNN models trained with data augmentation. Overall, MET-PET showed a higher predictive performance than T1c-w MRI for both endpoints and the DenseNet performed best (test data, TTR: C-index 0.66, OS: C-index 0.64). For both endpoints, the DenseNet prediction led to significant patient stratifications into risk groups (TTR: *p* 0.027, OS: *p* 0.033). Integrating these models with the clinical parameters age and MGMT status slightly improved their performance (test data, TTR: C-index 0.68, OS: C-index 0.65) and patient stratification was still significant (TTR: *p* 0.017, OS: *p* 0.039). Figure 4 shows the Kaplan–Meier curves for the best performing Clinical + DenseNet model for prognosis of TTR (a) and OS (b) using MET-PET imaging. The corresponding calibration plots are shown in Supplementary Fig. S9.

**Table 4:** Ensemble concordance index (C-index) values for cross validation (CV) on the training and test data for TTR and OS prediction based on MET-PET and T1c-w MRI data using deep learning (DL).

Endpoint	Modality	Model	C-index train	C-index valid	C-index test	*p*-value test
TTR	MET-PET	DenseNet	**0.84 (0.79–0.88)**	**0.68 (0.60–0.75)**	**0.66 (0.51–0.81)**	**0.027**
		ResNet	0.90 (0.85–0.93)	0.63 (0.56–0.71)	0.61 (0.43–0.79)	0.168
		VGGNet	0.84 (0.79–0.89)	0.69 (0.62–0.76)	0.55 (0.44–0.67)	0.763
	T1cw-MRI	DenseNet	0.86 (0.82–0.90)	0.63 (0.56–0.71)	0.50 (0.43–0.58)	0.406
		ResNet	0.82 (0.78–0.85)	0.60 (0.51–0.70)	0.55 (0.46–0.64)	0.096
		VGGNet	0.66 (0.60–0.73)	0.53 (0.46–0.60)	0.56 (0.45–0.68)	0.857
	Clinical + DenseNet MET-PET	**Cox**	**0.85 (0.81–0.88)**	**0.74 (0.67–0.79)**	**0.68 (0.53–0.83)**	**0.017**
OS	MET-PET	DenseNet	**0.82 (0.77–0.87)**	**0.61 (0.53–0.69)**	**0.64 (0.43–0.86)**	**0.033**
		ResNet	0.87 (0.84–0.91)	0.55 (0.47–0.62)	0.61 (0.44–0.77)	0.227
		VGGNet	0.88 (0.82–0.93)	0.70 (0.64–0.76)	0.53 (0.42–0.65)	0.426
	T1cw-MRI	DenseNet	0.84 (0.80–0.89)	0.62 (0.55–0.69)	0.60 (0.43–0.77)	0.067
		ResNet	0.87 (0.82–0.92)	0.58 (0.50–0.65)	0.59 (0.49–0.70)	0.191
		VGGNet	0.59 (0.51–0.66)	0.49 (0.42–0.57)	0.65 (0.55–0.76)	–
	Clinical + DenseNet MET-PET	**Cox**	**0.82 (0.77–0.87)**	**0.69 (0.63–0.75)**	**0.65 (0.51–0.78)**	**0.039**

Significant values are in [bold].

## Discussion

We investigated conventional radiomics and deep-learning-based radiomics (3D-CNNs) for detection of residual tumour status and to prognosticate TTR and OS in patients with newly diagnosed GBM based on MET-PET and T1c-w MRI. Overall, classification of residual tumour status and prognosis of TTR and OS on MET-PET was possible with a higher accuracy than on T1c-w MRI. In terms of modelling, the best performance in independent test data for detection of residual tumour status on MET-PET was achieved by the 3D-DenseNet (AUC 0.95), while logistic regression using conventional radiomics features performed best for T1c-w MRI (AUC 0.78). For prognosis of TTR and OS, the best performance on the independent test data was achieved by combining a clinical signature (age and MGMT) with a 3D-DenseNet ensemble model based on MET-PET imaging, with significant stratification of the patients in a low and high-risk group.

Several studies have examined the utilization of radiomics and automated algorithms for the purpose of diagnostic and prognostic modelling in GBM. Given that MRI currently represents the most extensively available imaging modality for GBM patient management, the majority of these studies have assessed the effectiveness of their proposed methods on MRI data. To the best of our knowledge, this is the first radiomics based evaluation of the diagnostic and prognostic role of pre-RCT [^11^C] MET-PET together with T1c-w MRI in adult patients with newly diagnosed GBM.

Commonly, the imaging-based assessment of the residual tumour status is done visually by experienced radiation oncologists, nuclear medicine experts, and radiologists in a complex evaluation procedure that is at risk for inter-rater variability^54^. In post-operative management of GBM, most of studies examined a semi-automated computer aided volumetry (CAV) approach for residual tumour detection on post-operative T1c-w MRI using small cohorts. For example, the studies by Kanaly et al.^55^ and Chow et al.^56^ demonstrated that a semi-automated CAV approach for residual tumour segmentation can reduce inter-observer variability. Among fully automated approaches for residual tumour segmentation, Meier et al.^25^ used an end-to-end machine learning based algorithm with a performance comparable to a human rater. Krivoshapkin et al.^57^ used an automated tool based on a mathematical model and showed that automatically measured residual tumour burden was a significant predictor of OS (*p*-value < 0.001). While our analysis did not involve segmentation, we were able to demonstrate the high performance of MET-PET in detecting residual tumour. This result can also be useful for segmentation algorithms by providing a reliable initialization method for identifying the target region of radiotherapy planning.

The use of radiomics analysis for prognostic modeling in GBM has been extensively investigated. However, it is worth noting that a large proportion of these studies have focused on pre-treatment mpMRI data as the primary imaging modality for analysis. Li et al.^58^, Kickingereder et al.,^59^ and Chaddad et al.^60^ showed that a prognostic model built with second order texture features extracted from pre-treatment mpMRI are significantly associated to OS in GBM (C-index 0.70^58^, C-index 0.65^59^, log-rank test *p*-value < 0.01^60^). Carles et al.^33^ and Manabe et al.^61^ observed that second-order texture features extracted from [18F]-FDG PET and MET-PET can predict OS (p 0.038^33^ and *p* < 0.05^61^, respectively). Verma et al.^62^ found higher order pre-treatment MRI features as a prognostic marker for PFS in GBM (C-index 0.80). Other studies have demonstrated that simple features (not strictly radiomics) extracted from pre-treatment MET-PET and MRI can also prognosticate OS in GBM^63–65^. A recent study by Garcia‑Ruiz et al.^31^ showed that a radiomics signature (first-order and second-order features) from the enhancing residual tumour region obtained by subtracting early post-surgical T1c-w from T1w MRI has prognostic value for predicting > 2-year OS status (AUC 0.71). In our conventional radiomics analysis, which involved the extraction of handcrafted features from pre-RCT T1c-w MRI data, we found that both first-order (intensity histogram) and second-order texture (distance zone matrix) features exhibited a notable correlation with overall survival (OS) in the training data. However, the signature was unable to demonstrate similar success when applied to the test data.

Studies have also demonstrated improved prognostic performance of patient clinical\molecular features when combined with radiomics features extracted from pre-treatment mpMRI. For the prediction of OS, Lao et al.^66^ showed that a model combining CNN-based deep features with clinical parameters (age and KPS) has improved prognostic performance than clinical features alone (C-index radiomics + clinical 0.74), while Tixier et al.^67^ showed that Gabor skewness features extracted from T1c-w MRI when combined with MGMT have improved prognostic performance compared to MGMT alone (log-rank p-value MGMT + radiomics 0.001). Similarly, Kickingereder et al.^59^ showed that combined clinical and radiomics model has better prognostic performance for OS prediction than individual models (C-index 0.69). Overall, the performance of our best performing conventional radiomics signature for the prognosis of OS based on features extracted from MET-PET and T1c-w MRI was somewhat lower (MET-PET C-index 0.60, T1c-w MRI C-index 0.63) than the results presented in literature. However, a full comparison with previous studies is not possible as we used post-surgical imaging (acquired after median 23 days) instead of pre-treatment imaging for prognostic modelling, where the tumour was mainly removed.

Further we also observed methodological heterogeneity found across the radiomics studies mainly due to the use of different software implementations and underreporting. This limitation is also highlighted in our recent study^68^. Thus, there is a strong need for a standard radiomics process for signature definition, both for reproducibility and progression of radiomics towards clinical application. To enhance the reliability of existing radiomics models, initiatives such as the IBSI^37^ are attempting to establish reporting guidelines for image processing and feature extraction. To tackle this problem, we have established and independently validated conventional radiomics signatures using the MIRP software, which is developed in accordance with the IBSI guidelines^37^, and we report parameters and algorithms used for their extraction, transformation, stability analysis, and modelling.

The lower performance of MRI-based classification in our radiomics analysis can be attributed to the clinical practice of assessing the extent of resection and tumour residual status in GBM through early post-operative MRI (within 24–48 h of surgery). Later MRI scans are susceptible to non-tumour-related contrast enhancement caused by inflammatory/repair-related changes, referred to as confounding effects. These changes can be mistaken for tumour remnants, leading to inaccurate diagnoses^69^. Consequently, using only the second baseline MRI for analysis, which was the only MRI available, is limited in terms of diagnostic accuracy. Since the residual tumour burden is a prognostic imaging biomarker in GBM^18,70^, misinterpretation of residual tumours can also lead to reduced prognostic performance. An example of such case is discussed in Supplementary Section 3 with Supplementary Fig. S10, showing a second baseline T1c-w MR image with confounding effects of surgically induced contrast enhancement. This contrast enhancement led to a misclassification of the T1c-w MR residual tumour status by the 3D-DenseNet model and indicates that the inclusion of early post-operative MRI may help to improve predictions. On the other hand, MET-PET is capable of providing better differentiation of nonspecific postoperative changes in GBM and therefore provides improved prognostic and diagnostic performance^71^.

In our review of relevant studies, as summarized in Table S13, numerous pre-treatment MRI-based radiomics investigations focus on the patient prognosis in GBM^58–60,62,64–67^. However, only a limited number of studies incorporate pre-treatment MET-PET with small sample sizes (N = 42^61^, N = 52^63^). Considering the significance of residual tumour burden as a prognostic imaging biomarker in GBM^18,19^, understanding the residual tumour status post-surgery may aid in refining treatment planning and tailoring therapies to the specific tumour burden, potentially improving treatment efficacy. Therefore, the radiomics based evaluation of post-operative data, in particular based on MET-PET imaging, may offer a better diagnostic and prognostic performance by providing clearer insights into the extent of residual disease. In this study, we included a relatively large data set of MET-PET imaging from a prospective clinical trial to establish the feasibility of such analysis. Further research is needed to evaluate the clinical applicability of proposed models.

In context of radiomics modelling, there is no ‘one size fit all’ scheme. Different machine learning and DL models have different performance, and the choice of an optimal model may not be immediately apparent. Therefore, trying multiple models can help to identify the model that best fits the data and achieves the highest performance. A study by Bae et al.^72^ compared conventional machine learning based radiomics with deep neural network (DNN) based radiomics analysis to distinguish GBM from brain metastases using pre-operative T2-w MRI and showed improved diagnostic value of the DNN compared to the best-performing machine learning model. Other studies have compared different machine learning models trained on radiomics features for differentiating gliomas^73,74^ and for prognostic modelling in other cancer entities^23^ and showed that model performance varies with algorithm used. In our radiomics analysis, we observed a limited gain from complex classifiers such as Xgboost_lm and RF compared to simple logistic regression, probably due to elaborate feature selection. Furthermore, we were able to show that CNNs, despite being highly parametrized models, were able to achieve a somewhat better performance than conventional radiomics. The improved performance of DL models can be attributed to the use of 3D-CNN models together with extensive data augmentation, as explained in the methodology of this study. Due to the volumetric nature of medical imaging, 3D-CNNs are more promising than 2D alternatives as they incorporate potentially relevant spatial information^75^. In our analysis, the performance benefit observed for residual tumour status detection and prognosis of TTR and OS on MET-PET via the 3D-DenseNet may be attributed to the use of multi-layer feature concatenation, which increases the representation capacity of CNNs. The generalizability of our 3D-CNN models was validated using an independent test cohort. Nevertheless, our conventional radiomics model outperformed the 3D-CNN model in predicting the status of residual tumour on T1c-w MRI, which is a commonly accessible modality for GBM. Thus, the choice between these models should depend on the specific requirements and availability of imaging data.

There are several open research questions concerning decision support and prognostication of outcome in newly diagnosed GBM that can be explored for future research with the help of DL. First, a prognostic model based on single-modality medical imaging only partially reflects the available tumour information. Similar to clinicians, who perform diagnoses and give prognostic suggestions, predictive models may be based on multimodal imaging data to extract more diverse aspects of phenotypical tumour information and integrate them in model development. In our conventional radiomics approach, we conducted such analyses, where we combined the final signatures developed from T1c-w MRI, MET-PET, and clinical/molecular features into a multivariable Cox model to predict the TTR and OS, however, leading to a similar performance as the best single models. One possible explanation for this result is that the lower performing T1c-w MRI may not add relevant information to the more reliable biomarkers from MET-PET imaging in the joint signature.

Prediction of the tumour recurrence location in GBM can enable more targeted and personalized therapies. Studies have investigated the use of machine learning based pattern recognition methods to provide predictive spatial maps of the early recurrence region using pre-treatment MRI^76,77^. However, additional research is needed to validate these findings and optimize the predictive models used for glioblastoma recurrence location prediction.

Our study has limitations. Even though the dataset used for this analysis is, so far, unique in the field of medical imaging, it contains a relatively low number of patients in the training and test cohorts, which may lead to model overfitting and wide confidence intervals. To overcome the problem of potential model overfitting, we used an extensive feature selection approach in conventional radiomics and data augmentation in the DL analysis. In addition, there is a small class imbalance in our tumour residual status detection analysis due to the smaller number of negative instances. We aimed to mitigate this problem by internal cross-validation (CV) on the training data for both conventional and DL based radiomics analysis. A fivefold CV approach was used and repeated 5 times, to ensure that each fold contained a sufficient number of negative instances for training and validation and that the finally considered ensemble model performance was sufficiently robust.

This study adheres to the 2016 WHO classification of brain tumours^78^, which categorizes IDH-mutant brain tumours as glioblastoma. However, in the updated 2021 WHO classification^79^, IDH-mutant tumours are no longer designated as glioblastoma but rather classified as astrocytoma, IDH-mutant. To assess the impact of this recent WHO classification on the generalizability of our results, we performed a re-validation of the radiomics signature, excluding IDH-mutant patients from the validation cohort. The re-validation showed only a minimal change of up to ± 2% in AUC and C-index, indicating the overall robustness of the proposed models.

In future work, we aim to validate our findings by additional datasets and adjust the present models if required. This approach will not only address the limitation of the currently relatively small dataset but may also contribute to a more balanced representation of tumour residual status and patient characteristics, thus eliminating disparities between the training and test cohorts. After additional prospective validation, our models may ultimately aid clinicians in diagnosis and prognosis, potentially reducing required resources, inter-rater variability, and facilitate the development of personalized radiotherapy.

In conclusion, we developed and independently tested conventional and DL-based radiomics for predicting the residual tumour status and prognosticate TTR and OS in patients with newly diagnosed GBM using MET-PET and T1c-w MRI acquired after surgery. Overall, residual tumour detection and prognosis on MET-PET was possible with a higher accuracy than on T1c-w MRI.

### Supplementary Information

Supplementary Information.

## Data Availability

The data that support the findings of this study are available on request from the corresponding author (S.L.). The data is not publicly available due to patient data privacy policy.
