# Supplementary material for: Radiomics for residual tumour detection and prognosis in newly diagnosed glioblastoma based on postoperative [11C] methionine PET and T1c-w MRI
Source: Sci Rep. 2024 Feb 25;14:4576. doi: 10.1038/s41598-024-55092-8 (PMC10894870; doi:10.1038/s41598-024-55092-8)
Supplement: Supplementary file 1 — Supplementary Information. [file 41598_2024_55092_MOESM1_ESM.pdf]

Supplementary material: Radiomics for the detection of residual tumour status after surgery and patient outcome prediction after chemoradiotherapy in newly diagnosed glioblastoma based on [^{11}C] methionine PET and T1c-w MRI

Iram Shahzadi, Annekatrin Seidlitz, Bettina Beuthien-Baumann, Alex Zwanenburg, Ivan Platzek, Jörg Kotzerke, Michael Baumann, Mechthild Krause1, Esther G.C. Troost, and Steffen Löck

Table S1: Image acquisition parameters of diagnostic magnetic resonance imaging (MRI) and positron emission tomography (PET) data for both training and test data.

**Imaging parameters MRI**		**Imaging parameters PET**	
**Pixel spacing / mm**	0.96-1.0	**Pixel spacing /mm**	1.0-2.0
**Slice thickness / mm**	1.0	**Slice thickness / mm**	2.0
**Flip angle / °**	8	**Field of view**	[903,180]
**Scanning sequence**	Gradient recall	**Attenuation Correction**	MARC
**Field strength / T**	3	**Reconstruction method**	LOR-RAMLA
**Scanner**	3T Ingenuity TF PET/MRI scanner (Philips Healthcare, Best, The Netherlands)	**Scanner**	3T Ingenuity TF PET/MRI scanner (Philips Healthcare, Best, The Netherlands)
		**Random correction method**	DLDYD
		**Scatter correction method**	SS-SIMUL
		**Acquisition duration**	40 min
		**MET bolus**	300 MBq +/- 53 MBq
		**Acquisition post injection**	20-40 min

Table S2: Feature classes extracted from MET-PET and T1c-w MRI. LoG transformations used for intensity-based features. fbs: fixed bin size, fbn: fixed bin number, MET: [11C] methionine, PET: positron emission tomography, IBSI: image biomarker standardization initiative, LoG: Laplacian of Gaussian

**Features**	**Number of features**	**Modality**	**IBSI Identifier**
(i) Local intensity features	2	PET / MRI	9ST6
(ii) Intensity-based statistical features	18	PET / MRI	UHIW
(iii) Intensity-volume histogram features	14	PET / MRI	P88C
(iv) Intensity histogram features fbn = 16	23	PET / MRI	ZVCW
(iv) Intensity histogram features fbs = 0.25	23	PET	ZVCW
(v) Texture features fbs = 0.25, fbn = 16		PET (fbs,fbn) / MRI (fbn)	
Grey level co-occurrence based features	25		LFYI
Grey level run length based features	16		TP0I
Grey level size zone based features	16		9SAK
Grey level distance zone based features	16		VMDZ
Neighbourhood grey tone difference based features	5		IPET
Neighbourhood grey level based features	17		REK0
(vi) Log transformed features (i)-(iv)	57	PET / MRI	
Total	PET=327<br>MRI=209		

Table S3: Image preprocessing parameters for both PET and MRI data, as used in MIRP.

Parameters	PET/MRI
Interpolated isotropic voxel spacing (mm)	2/1
Image interpolation method	linear
ROI interpolation method	linear
Merge method for texture matrices	volume merge (IBSI: IAZD and KOBO)
Discretisation method: fixed bin number (bins)	16/32
Discretisation method: fixed bin size (bin width)	0.25/ –
Laplacian of Gaussian sigma	2 mm/1 mm

Section 1: Feature selection criteria for final signature

Here we present an example of feature selection for residual tumour status prediction on MET-PET imaging. The same technique applies to residual tumour status prediction on T1c-w MRI as well. 39 MET-PET features with the highest mutual information (measured by the AUC) with residual tumour status on MET-PET were selected after hierarchical clustering. These features were then used to build a diagnostic model. Feature selection and model building with internal validation was first performed within 5 repetitions of 5-fold cross-validation (CV) nested in the training data to identify an optimal signature, with model performance evaluated in terms of median AUC across all CV folds. For each of the above-mentioned feature selection methods, the occurrence of every feature in the 25 modelling steps (5 repetitions of 5-fold CV) was counted, and features were ranked according to their occurrences across the cross-validation folds. Table S4 shows features with top 5 ranks across each feature selection method that were further considered. Finally, features that showed repeated occurrences across at least 75% of the feature selection methods were selected. Two features, log_ih_kurt_fbn_n16 and log_stat_skew, occurred in all 4 feature-selection methods, thus meeting the 75% occurrence criteria for candidate features. Both features showed a Spearman correlation of >0.5 on the entire training data as shown in Figure S1(a). Finally, log_ih_kurt_fbn_n16 was selected as a one-feature signature due to the stronger association of this feature with the endpoint (p=2.58×10^{-5}) as compared to log_stat_skew (p=8.37×10^{-5}). The finally selected signature and the average AUC (average of AUC across all feature selection methods) in internal training and external test are reported in the results section. The same technique is applicable to the detection of residual tumour status on T1-c w MRI and for the prognosis of TTR (example shown in Table S5) and OS.

Table S4: Median AUC for PET-status prediction based on MET-PET data using cross-validation of the training data with logistic regression. Top 5 features ranked according to their occurrence are shown here. Features with a repeated occurrence across at least 75% (3 out of 4) of the feature selection methods are marked in bold. AUC: area under the curve, CV: cross-validation, EN: elastic net, MRMR: minimum redundancy maximum relevance, MIM: mutual information maximization, UR: univariate regression.

Modality	Feature selection	CV training AUC	CV validation AUC	Features	Rank	Selected features
**PET**	**MRMR**	0.94	0.90	stat_rms<br>**log_ih_kurt_fbn_n16**<br>ngl_dcnu_norm_d1_a0_0_3d_fbn_n16<br>log_loc_peak_loc<br>**log_stat_skew**	1<br>2<br>3<br>4<br>5	**log_ih_kurt_fbn_n16**<br>**log_stat_skew**<br><br>Remarks: Both features occurred in at least 3 out of 4 (75%) feature selection methods. These features showed a correlation >0.5 (Figure S1a). Finally, **log_ih_kurt_fbn_n16** was selected as a signature due to stronger association with the endpoint compared to **log_stat_skew.** **log_ih_kurt_fbn_n16** was used **to** build final models using logistic regression (GLM_logistic), Xgboost linear model (XGB_lm) and random forest (RF) learners.
	**MIM**	0.95	0.93	stat_rms<br>**log_ih_kurt_fbn_n16**<br>ngl_dcnu_norm_d1_a0_0_3d_fbn_n16<br>log_loc_peak_loc<br>**log_stat_skew**	1<br>2<br>3<br>4<br>5	
	**UR**	0.95	0.93	**log_ih_kurt_fbn_n16**<br>log_stat_min<br>log_ih_cov_fbn_n16<br>dzm_sdhge_3d_fbn_n16<br>**log_stat_skew**	1<br>2<br>3<br>4<br>5	
	**EN**	0.96	0.94	dzm_sdhge_3d_fbn_n16<br>ivh_v75<br>**log_ih_kurt_fbn_n16**<br>dzm_zdnu_norm_3d_fbn_n16<br>**log_stat_skew**	1<br>1<br>2<br>3<br>4	
**Average AUC**		0.95	0.93			Average AUC is reported in Table 2 for radiomics performance

Table S5: Median C-index for prognosis of TTR based on MET-PET data using cross-validation of the training data with Cox regression. Top 5 features ranked according to their occurrence are shown here. Features with a repeated occurrence across at least 75% (3 out of 4) of the feature selection methods are marked in bold. CI: concordance index, CV: cross-validation, EN: elastic net, MRMR: minimum redundancy maximum relevance, MIM: mutual information maximization, TTR: time-to-recurrence, UR: univariate regression.

Modality	Feature selection	CV training C-index	CV validation C-index	Features	Rank	Selected features
**PET**	**MRMR**	0.66	0.59	**dzm_sdhge_3d_fbn_n16**<br>log_stat_mean<br>**log_stat_min**<br>ngl_glnu_d1_a0_0_3d_fbn_n16<br>**log_ivh_i90**	1<br>1<br>3<br>4<br>5	**dzm_sdhge_3d_fbn_n16**<br>**log_stat_min**<br>**log_ivh_i90**<br>**log_ih_skew_fbn_n16**<br><br>Remarks: **dzm_sdhge_3d_fbn_n16, log_stat_min, log_ivh_i90** and **log_ih_skew_fbn_n16** occurred in at least 3 out of 4 (75%) feature selection methods. All these features showed a correlation >0.5 (Figure S1b). Finally **log_stat_min** was selected due to stronger association with the endpoint compared to other features, and was used to build final models using Cox regression (Cox), Xgboost linear model (XGB_lm) random survival forest (RSF) learners.
	**MIM**	0.66	0.58	**log_stat_min**<br>**dzm_sdhge_3d_fbn_n16**<br>**log_ivh_i90**<br>ngl_glnu_d1_a0_0_3d_fbn_n16<br>**log_ih_skew_fbn_n16**	1<br>2<br>3<br>4<br>5	
	**UR**	0.66	0.60	**log_stat_min**<br>**dzm_sdhge_3d_fbn_n16**<br>**log_ivh_i90**<br>rlm_rl_var_3d_v_mrg_fbn_n16<br>**log_ih_skew_fbn_n16**	1<br>1<br>3<br>4<br>5	
	**EN**	0.67	0.60	**dzm_sdhge_3d_fbn_n16**<br>**log_ivh_i90**<br>**log_stat_min**<br>**log_ih_skew_fbn_n16**<br>stat_rms	1<br>2<br>2<br>3<br>4	
**Average CI**		0.66	0.59			Average CI is reported in Table 4 for radiomics performance

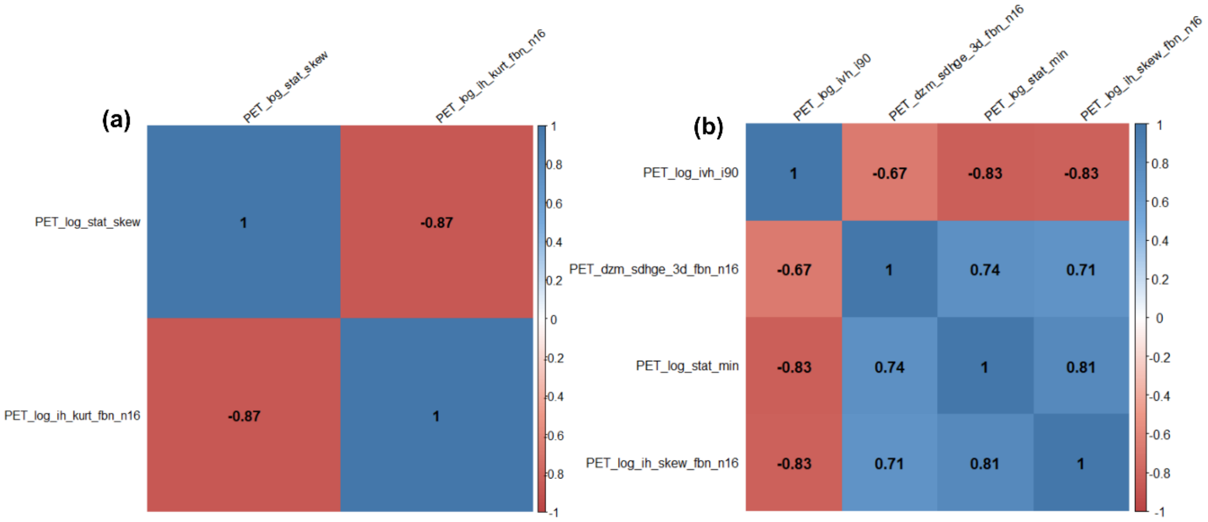

Figure S1: Correlation plot of features with a repeated occurrence across at least 75% (3 out of 4) of the feature selection methods (a) for prediction of residual tumour status on MET-PET, and (b) for prognosis of TTR. Features showed a high correlation ($\rho > 0.5$).

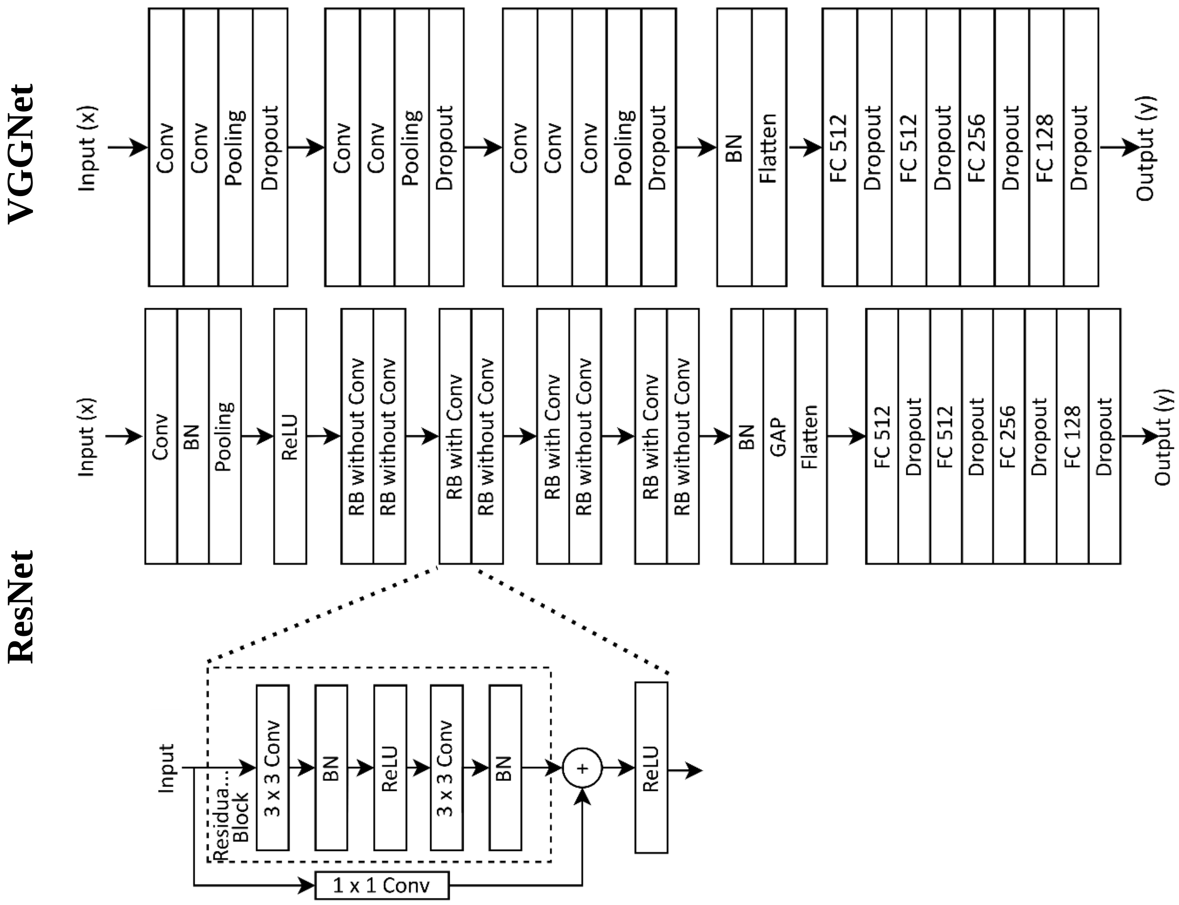

Figure S2: An illustration of 16-layer VGGNet and 18-layer ResNet with its building block, i.e. residual block with 1 × 1 convolution. Residual blocks with and without 1 × 1 convolution are stacked together to form the ResNet architecture. BN: batch normalization, Conv: convolution layer, GAP: global average pooling layer, FC: fully connected layer, RB: residual block.

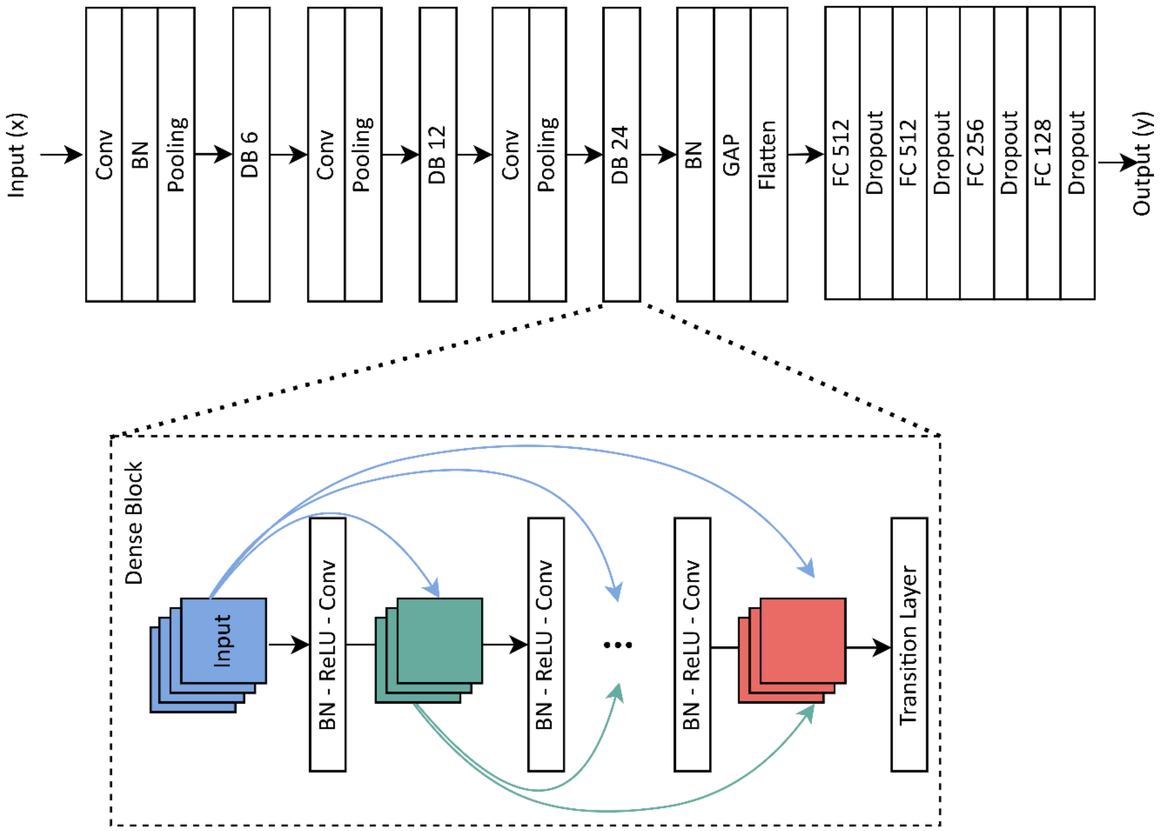

Figure S3: An illustration of DenseNet and its building block, i.e. dense block. In each dense block, output from each convolution layer is combined with output of all subsequent layers within the desne block. BN: batch normalization, Conv: convolution layer, DB: dense block, FC: fully connected layer, GAP: Global average pooling.

Section 2: 3D data augmentation

In this work, we used random flipping to create mirror reflections of the input image volume along only the in-plane x and y axis. Mirroring in batchgenerators is evenly distributed and probability of mirroring along each axis is 0.5. The rest of augmentations used in this work belong to pixel-level transformations. We used additive Gaussian noise with variance uniformly sampled from the range (0, 0.05). We also used Gaussian blur with standard deviation (σ) selected randomly from the range (1, 1.75). Further, we used Gamma correction to improve luminance of input volumes with gamma values selected randomly from the range (0.5, 2). Finally, brightness multiplicative transform, where the multiplier is randomly sampled from range of (0.7, 1.5), and random contrast transform, where contrast values were randomly sampled from the interval (1, 1.75), were used for augmenting T1c-w MRI data only. We did not use brightness and contrast transform for MET-PET data, as the effect of these transformation was found to be less effective for improving model performance. The hyperparameters for pixel-level transforms were selected manually by visually inspecting the images so that each transformation creates an image that is representative of real perturbations and by avoiding extreme transformations with very high or low values of transformation parameters. All transformations were applied to 3D patches extracted around the clinical target volume (CTV). Each augmentation was applied with the probability of 0.15 which limits the number of original images shown to the network. The percentage of original images used during the training was 40% and 10%, combining 4 and 6 different augmentation techniques for MET-PET and T1c-w MRI, respectively.

Table S6: Data augmentation parameters used for deep learning analysis. Augmentations were carried out using the batchgenerators package, which is an open-source python package for data augmentations.

**Augmentation**	**Modality**	**Parameters**
Mirror	PET/MRI	Axes = (0, 1)
Gamma transform	PET/MRI	gamma range = (0.5, 2)
Gaussian noise	PET/MRI	Noise variance = (0, 0.05)
Gaussian blur	PET/MRI	Blur sigma = (0.5, 1.5)
Contrast	MRI	Range = (1, 1.75)
Brightness multiplicative transform	MRI	Range = (0.7, 1.5)

Table S7: Univariable analysis of time-to-recurrence (TTR), and overall survival (OS) using Cox regression, in the training data. ci: confidence interval. Significant p-values of patient's clinical characteristics are marked in bold.

**Clinical feature**		**TTR**		**OS**	
		**Hazard ratio (95% ci)**	**p-value**	**Hazard ratio (95% ci)**	**p-value**
**Age** / years		1.017 (1.001-1.033)	**0.034**	1.028 (1.011-1.045)	**0.001**
**Gender** (female vs male)		1.085 (0.679-1.733)	0.733	0.919 (0.57-1.481)	0.728
**MGMT** (methylated vs wildtype)		0.251 (0.147-0.429)	**<0.001**	0.251 (0.144-0.437)	**<0.001**
**ECOG**	(1 vs 0)	1.135 (0.705-1.826)	0.603	1.147 (0.702-1.873)	0.584
	(2 vs 0)	1.624 (0.635-4.149)	0.311	2.243 (0.868-5.796)	0.095
**IDH** (mutated vs wildtype)		0.243 (0.076-0.78)	**0.018**	0.319 (0.099-1.022)	**0.054**
**PET status (0 vs 1)**		2.578 (1.518-4.379)	<0.001	2.119 (0.472-1.251)	0.005
**MRI status (0 vs 1)**		2.755 (1.717-4.421)	<0.001	2.129 (1.331-3.406)	0.002
Abbreviations: ECOG, Eastern Co-operative Oncology Group; IDH, isocitrate dehydrogenase; MGMT, O6-methylguanine DNA methyltransferase; MRI, magnetic resonance imaging; OS, overall survival; PET, positron emission tomography; TTR, Time-to-recurrence.					

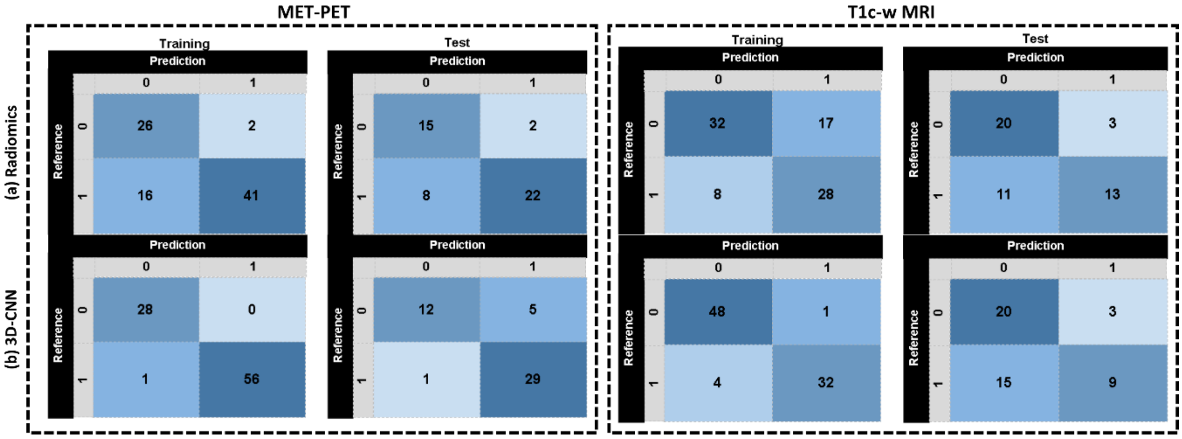

Figure S4: Confusion matrices for residual status prediction in training and test data based on MET-PET and T1c-w MRI data (a) using the final radiomics-based logistic regression model and (b) using the final 3D-DenseNet model.

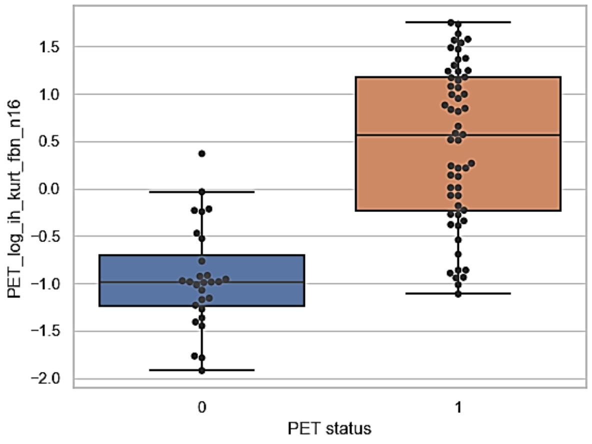

Figure S5: Box plot of Yeo-Johnson transformed and z-score normalized features selected in the best performing MET-PET signature for prediction of residual tumour status on MET-PET in training data. PET_log_ih_kurt_fbn_16 showed relatively higher values in MET-PET positive patients as compared to MET-PET negative patients.

Table S8: Summary of the selected T1c-w MRI-based radiomics signature for residual tumour status detection on T1c-w MRI. GLDZM: grey level size zone matrix, IH: intensity histogram.

Modality	Features	Identifier	Feature type	Definition
**MRI**	dzm_ldhge_3d_fbn_n32	KLTH	GLDZM	This feature emphasizes runs in the lower right quadrant of the GLDZM, where large zone distances and high grey levels are located. In essence, core regions with high intensity.
	Ih_rmad_fbn_n32	WRZB	IH	The mean absolute deviation is a measure of dispersion from the mean of discretized intensities.

Table S9: Final models for the residual tumour status on MET-PET and T1c-w MRI using conventional radiomics. Training was performed on the entire training cohort using multivariable logistic regression. In addition, transformation parameters from the Yeo-Johnson transformation and z-normalization, and optimal cutoff values from Youden's index are given.

Modality	Feature	Coefficient	p-value	Yeo-Johnson (λ)	z-score normalization (mean, sigma)	Cutoff
**PET**	log_ih_kurt_fbn_n16<br>intercept	2.65<br>1.67	<0.001<br>-	-0.3<br>-	(1.14, 0.52)<br>-	0.77
**MRI**	dzm_ldhge_3d_fbn_n32<br>ih_rmad_fbn_n32<br>intercept	1.15<br>-0.64<br>-0.39	0.001<br>0.05<br>-	-0.3<br>-1.4<br>-	(3.14, 0.04)<br>(0.52, 0.04)<br>-	0.38

Table S10: Ensemble AUC values for training and internal validation CV folds for PET status prediction based on MET-PET imaging and MRI status prediction based on T1c-w MRI data using deep learning with and without data augmentation. Models trained with data augmentation showed relatively higher performance in internal validation, compared to models trained without data augmentation. AUC: area under the curve, CV: cross validation, MET: 11C methionine, PET: positron emission tomography, MRI: magnetic resonance imaging.

Modality	Model	CV train AUC		CV valid AUC	
		Without augmentation	With augmentation	Without augmentation	With augmentation
MET-PET	DenseNet	1.00	1.00	0.88	0.96
	ResNet	1.00	1.00	0.86	0.92
	VGG	1.00	1.00	0.96	0.95
T1c-w MRI	DenseNet	0.87	1.00	0.61	0.77
	ResNet	0.91	1.00	0.61	0.73
	VGG	0.82	0.99	0.66	0.71

Table S11: Ensemble C-index values for training and internal validation for prognosis of TTR and OS based on MET-PET imaging and T1c-w MRI data using deep learning with and without data augmentation. Overall, models trained with data augmentation showed higher performance in internal validation, compared to models trained without data augmentation. C-Index: concordance index, CV: cross validation, MET: 11C methionine, PET: positron emission tomography, MRI: magnetic resonance imaging.

**Modality**	**Model**	**CV train C-index**		**CV validation C-index**	
		**Without augmentation**	**With augmentation**	**Without augmentation**	**With augmentation**
MET-PET	DenseNet	0.75	0.84	0.68	0.68
	ResNet	0.84	0.90	0.62	0.63
	VGGNet	0.85	0.84	0.66	0.69
T1c-w MRI	DenseNet	0.87	0.86	0.59	0.63
	ResNet	0.82	0.82	0.57	0.60
	VGGNet	0.55	0.66	0.60	0.53
MET-PET	DenseNet	0.63	0.82	0.59	0.61
	ResNet	0.89	0.87	0.58	0.55
	VGGNet	0.87	0.88	0.68	0.70
T1c-w MRI	DenseNet	0.88	0.83	0.62	0.64
	ResNet	0.87	0.87	0.59	0.58
	VGGNet	0.72	0.59	0.53	0.49

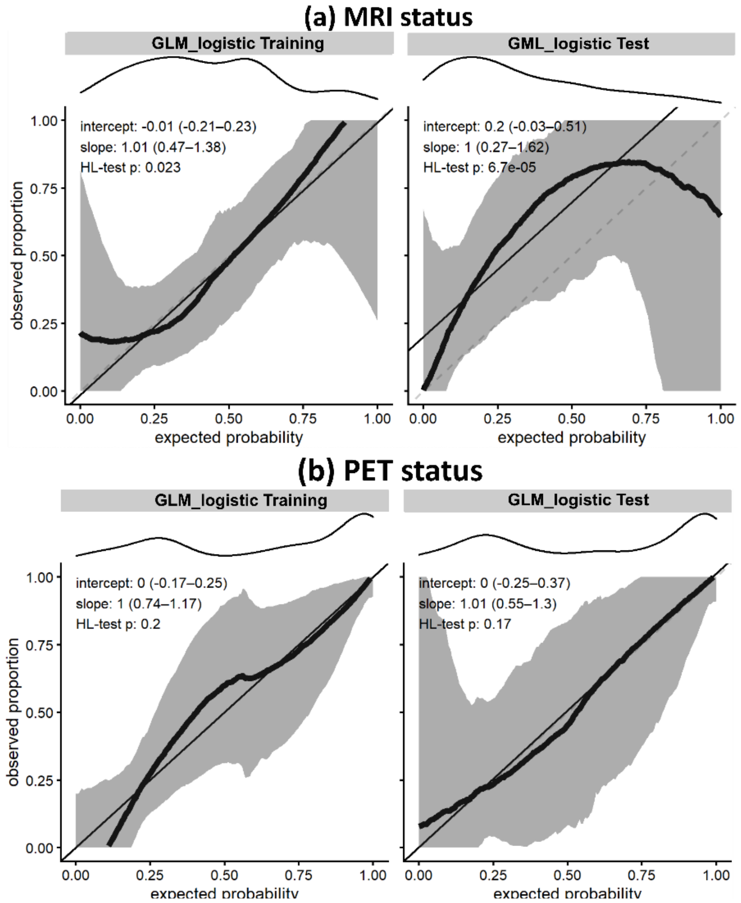

Figure S6: Calibration plots on training and test data for the residual tumour status detection in (a) T1c-w MRI, and (b) MET-PET using conventional radiomics. For calibration, data (thick lines) and 95% confidence intervals (shaded regions) are shown together with linear regression lines (solid lines) and optimal expectation (dashed lines). Density of expected probabilities is shown above the calibration plot. The residual tumour status detection on MET-PET shows good fit of prediction data.

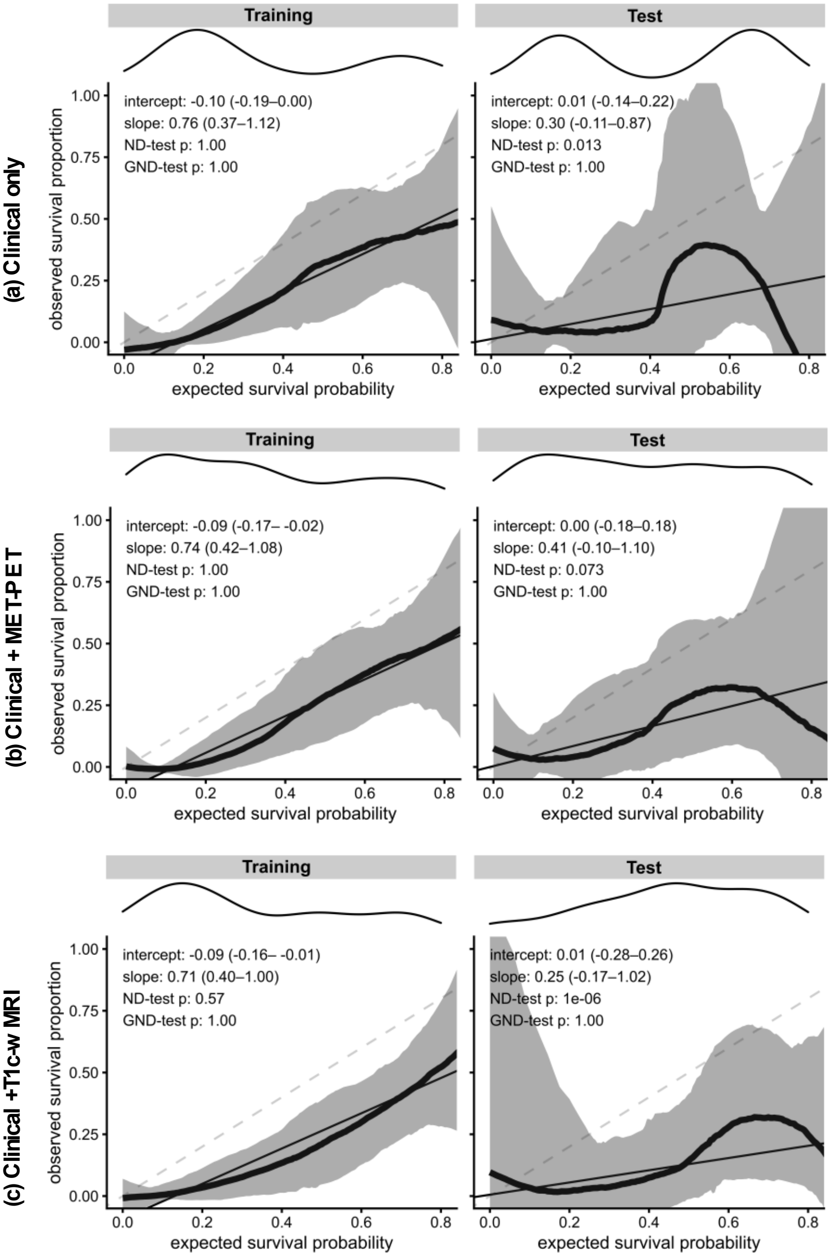

Figure S7: Calibration plots on training and test data for the prognosis of TTR (after 5 years) using the Cox regression model based on (a) clinical only signature, (b) clinical + MET-PET signature and (c) clinical + T1c-w MRI signature. For calibration, data (thick lines) and 95% confidence intervals (shaded regions) are shown together with linear regression lines (solid lines) and optimal expectation (dashed lines). Density of expected probabilities is shown above the calibration plot.

Table S12: Final model coefficients for the prognosis of TTR and OS using the clinical only, the clinical + MET-PET and the clinical + MRI radiomics models. Training was performed on the entire training data using multivariable logistic regression. In addition, transformation parameters from the Yeo-Johnson transformation and z-normalization, and optimal cutoff values for Kaplan Meier plots are presented.

Endpoint	Signature	Features	Coefficient	p-value	Yeo-Johnson (λ)	z-score normalization (mean, sigma)	Cutoff
TTR	Clinical	MGMT	0.23	<0.001	-	-	0.53
		Age / years	1.32	0.015	1.9	1195.451, 489.816	
	Clinical + PET	MGMT	0.26	<0.001	-	-	0.62
		Age / years	1.31	0.019	1.9	1195.451, 489.816	
		log_stat_min	0.70	0.004	10	-0.043, 0.018	
	Clinical + MRI	MGMT	0.22	<0.001	-	-	0.40
		Age / years	1.32	0.037	1.9	1195.451, 489.816	
		ivh_diff_i25_i75	0.76	0.031	-0.4	2.109, 0.039	
		dzm_zd_var_3d_fbn_n32	1.44	0.008	0	3.680,0.512	
		loc_peak_glob	1.12	0.354	0.2	1.163,0.272	
OS	Clinical	MGMT	0.20	<0.001	-	-	0.58
		Age / years	1.61	<0.001	1.9	1195.451, 489.816	
	Clinical + PET	MGMT	0.21	<0.001			0.68
		Age / years	1.60	<0.001	1.9	1195.451, 489.816	
		stat_max	1.31	0.038	-4.4	0.140, 0.029	
	Clinical + MRI	MGMT	0.19	<0.001	-	-	0.46
		Age / years	1.72	<0.001	1.9	1195.451, 489.816	
		ivh_diff_i25_i75	0.79	0.046	-0.4	2.109, 0.039	
		dzm_zd_var_3d_fbn_n32	1.46	0.004	0	3.680, 0.512	

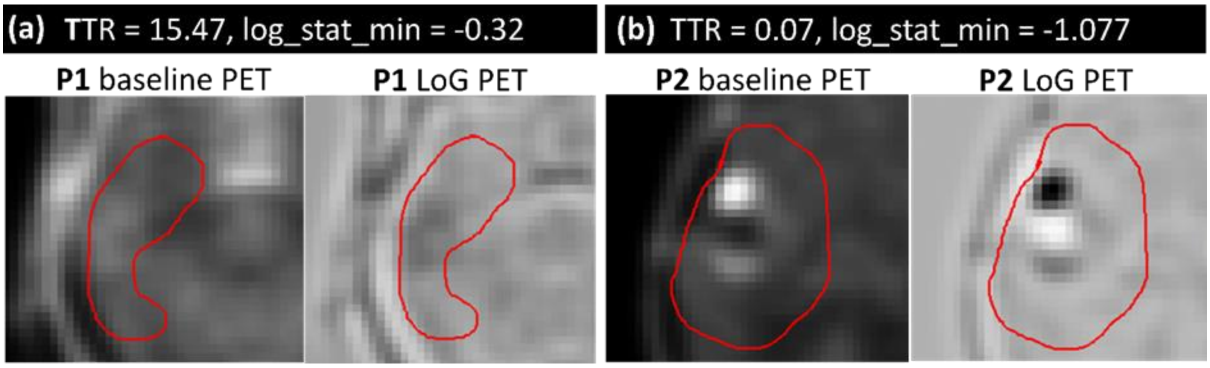

Figure S8: Representative images from MET-PET imaging with corresponding Laplacian of Gaussian (LoG) transformed images and the selected signature, i.e. log_stat_min value from two patients (P) in the two risk groups (P1 from low risk group and P2 from high risk group) of training data. The red contours mark the clinical target volume (CTV). P1 (TTR status = 0, TTR = 15.47 months) showed an overall homogenous appearance on the baseline MET-PET with higher log_stat_min value (a). On the contrary, P2 (TTR status = 1, TTR = 0.07 months) showed a more heterogeneous CTV with a low log_stat_min, which corresponds to high pixel intensities (stat_max) on the baseline PET (b).

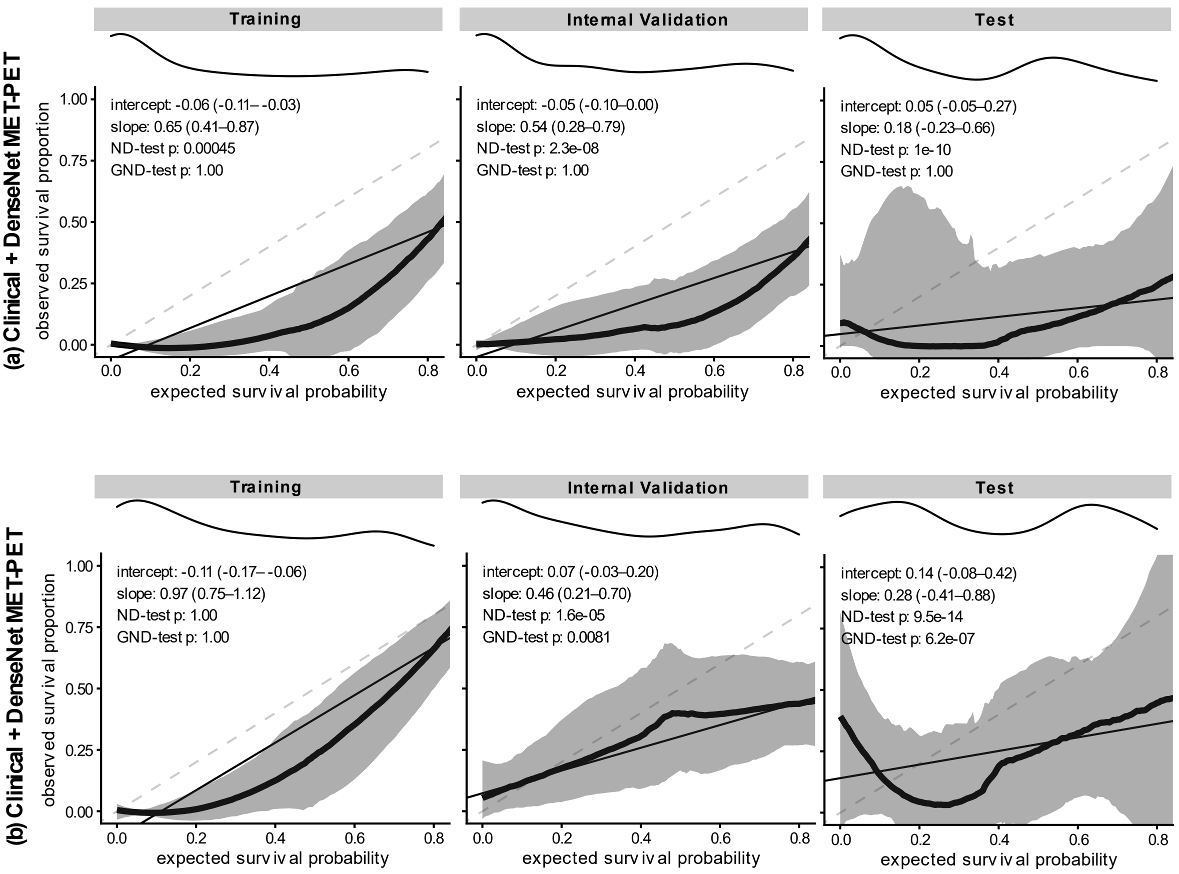

Figure S9: Calibration plots on training and test data for (a) time to recurrence (TTR) and, (b) overall survival (OS) in training, internal validation and external test data based on the respective joint clinical + ensemble predictions (3D-DenseNet model) on MET-PET data. For calibration, data (thick lines) and 95% confidence intervals (shaded regions) are shown together with linear regression lines (solid lines) and optimal expectation (dashed lines). Density of expected probabilities is shown above the calibration plot.

Section S3: Reasoning for misclassification of DenseNet model on MET-PET and T1c-w MRI

For MET-PET, the best performing DenseNet model showed a high sensitivity but lower specificity. We visually assessed false-positive predictions on the test data and observed that MET uptake in falsely classified images exhibit a greater asymmetry in intensity compared to the contralateral hemisphere, possibly due to infiltrative invasion of tracer in white matter (Figure S9(a)). However, MET-negative volumes used in training had an overall homogenous appearance with absence of asymmetry in intensity compared to the contralateral hemisphere (Figure S9(b)), making neural networks blind to images with greater asymmetry in intensity during training, which may lead to false-positive classifications in test data.

An example of misclassification due to post-surgical confounding effects in T1c-w MRI is shown in Figure S9(c-d). Figure S9(c) shows an image with confounding effects of surgically induced contrast enhancement. This contrast enhancement led to a misclassification of the T1c-w MR residual tumour status by the 3D-DenseNet model. The true clinical decision of MR-negative status was made on early post-surgical MRI shown in Figure S9(d), which does not show contrast enhancement on surgical boundaries. This indicates that the inclusion of early post-operative MRI may help to improve predictions.

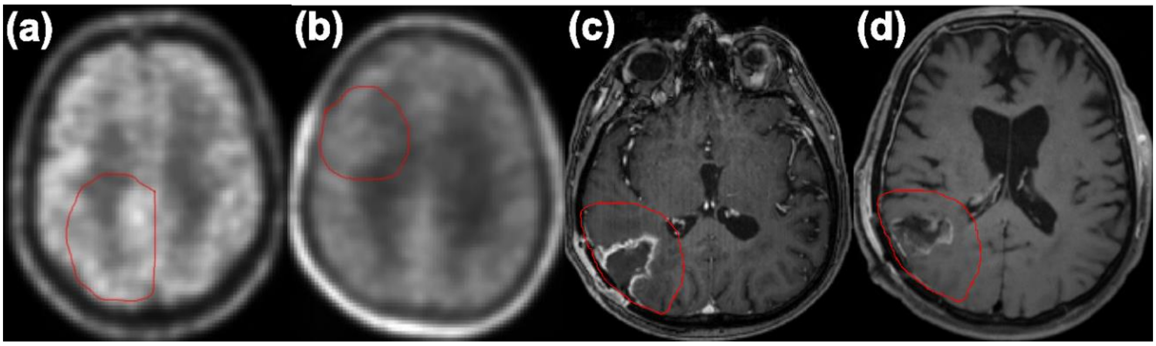

Figure S10: (a) Example PET image of a false-positive case. The PET image appears patchy, compared to (b) a true negative PET image with overall homogenous appearance. (c) Example T1c-w MR image of a false-positive case with confounding effects of surgically induced contrast enhancement in the second baseline MR image. The clinical decision of MR-negative status was made on early postsurgical MRI shown in (d), which does not show contrast enhancement on surgical boundaries. Red contours represent the CTV.

Table S13: Previous studies for prognostic modelling in glioblastoma. C-index: Concordance index, OS: overall survival, PFS: progression free survival.

Study	Imaging	Endpoint	Model/analysis	Features	Performance results
Krivoshapkin et al. [58]	Pre and Post-surgery T1 MRI	OS	Kaplan–Meier log-rank test	Residual Tumour volume	p-value <0.01
Li et al. [59]	Pre-treatment mpMRI	OS	Cox regression	Second-order	C-index 0.70
Kickingereder et al. 2016 [60]	Pre-treatment mpMRI	OS, PFS	Supervised Principal Component Analysis	Second-order	OS C-index 0.65, PFS C-index 0.61
Chaddad et al. 2018 [61]	Pre-treatment T1, FLAIR MRI	OS	Kaplan–Meier log-rank test	Second-order	p-value<0.01
Carles et al. 2021 [62]	Pre-treatment FDG-PET	OS	Kaplan–Meier log-rank test	Second-order	p-value 0.038
Manabe et al. 2021 [63]	Pre-treatment MET-PET	OS	Kaplan–Meier log-rank test	Second-order	p-value < 0.05
Verma et al. [64] 2020	Pre-treatment T1, FLAIR MRI	PFS	LASSO Cox	Higher order	C-index 0.80
Kobayashi et al. 2015 [65]	Pre-treatment MET-PET	OS	Kaplan–Meier log-rank test	Volume based MET features	p-value < 0.05
Pérez-Beteta et al. 2018 [66]	Pre-treatment T1 MRI	OS	Cox regression	Volume base MRI features	C-index 0.74
Gutman et al. 2013 [67]	Pre-treatment T1, FLAIR MRI	OS	Cox regression	Morphological	p-value 0.02
Garcia-Ruz et al. 2021 [31]	Post-surgical T1w MRI	OS	Logistic regression	First-order and Second-order	AUC 0.71
Kickingereder et al. 2016 [60]	Pre-treatment mpMRI	OS, PFS	Supervised Principal Component Analysis	Second-order and clinical	OS C-index 0.69, PFS C-index 0.64
Lao et al. 2017 [68]	Pre-treatment mpMRI	OS	Cox-regression	Deep feature and clinical	C-index 0.74
Tixier et al. 2019 [69]	Pre-treatment T1w	OS	Kaplan–Meier log-rank test	Second order and molecular	p-value < 0.01
